# Keto-Adamantane-Based Macrocycle Crystalline Supramolecular Assemblies Showing Selective Vapochromism to Tetrahydrofuran

**DOI:** 10.3390/molecules29030719

**Published:** 2024-02-04

**Authors:** Zunhua Li, Yingzi Tan, Manhua Ding, Linli Tang, Fei Zeng

**Affiliations:** College of Chemistry and Bioengineering, Hunan University of Science and Engineering, Yongzhou 415199, China; zhli@huse.edu.cn (Z.L.); shary8485034@126.com (Y.T.); lyn870807@126.com (L.T.)

**Keywords:** THF, self-assembly, vapochromism, CT cocrystals, supramolecular chemistry

## Abstract

Here, we report the synthesis of adamantane-based macrocycle **2** by combining adamantane building blocks with π-donor 1,3-dimethoxy-benzene units. An unpredictable keto-adamantane-based macrocycle **3** was obtained by the oxidation of **2** using DDQ as an oxidant. Moreover, a new type of macrocyclic molecule-based CT cocrystal was prepared through exo-wall CT interactions between **3** and DDQ. The cocrystal material showed selective vapochromism behavior towards THF, specifically, among nine volatile organic solvents commonly used in the laboratory. Powder X-ray diffraction; UV-Vis diffuse reflectance spectroscopy; ^1^H NMR; and single crystal X-ray diffraction analyses revealed that color changes are attributed to the vapor-triggered decomplexation of cocrystals.

## 1. Introduction

Organic charge transfer (CT) assemblies, which consist of donor (D) and acceptor (A) components, have received considerable attention due to their potential application in optoelectronics [1,2,3,4,5,6,7,8]; the pharmaceutical industry [9,10,11,12]; thermochromic/vapochromic materials [13,14,15,16,17,18,19]; and other areas [20,21,22,23]. The synthesis of single-molecule D-A assemblies based on intramolecular interactions are often complicated and challenging, and CT affinity between donors and acceptors in the intermolecular CT assemblies is frequently relatively weak [24,25,26,27,28]. Interactions between the components of macrocyclic molecule-based assemblies are relatively strong because of the presence of multiple auxiliary weak interactions, including π-π stacking, hydrogen bonding, electrostatic, and hydrophobic interactions, facilitating the preparation and enhancing the CT affinity of organic CT assemblies [29,30,31]. These macrocyclic molecule-based crystalline CT assemblies, which have stimuli-responsive properties, can be prepared in large scale in a modular approach. Because of this, many researchers are thus focusing on the construction of macrocyclic host-based crystalline CT assemblies [32,33,34].

Pillar[n]arenes have been used to produce crystalline CT assemblies owing to their ease of preparation and structural functionalization, easy formation of crystalline assemblies, and excellent adsorptive properties of the cavity towards guest molecules [13,14,15,16,17]. During the past two decades, various synthetic macrocyclic hosts have been reported one after another, like bamboo shoots after a spring rain [35,36,37,38,39,40,41,42]. However, besides pillar[n]arenes, crystalline macrocyclic molecule-based CT cocrystal assemblies are relatively unexplored [43]. Undoubtedly, the construction of macrocyclic molecules that can be easily produced, and can form crystalline CT assemblies with specific vapors in a short time, are called for but greatly challenging.

As for the fabrication of macrocyclic molecule-based CT cocrystals using new synthetic macrocyclic molecules, the following conditions should be satisfied. Firstly, π-donor or π-acceptor groups should be present in the macrocyclic molecules. Secondly, the macrocyclic molecule should easily form crystallized CT assemblies with guest molecules in the solid state through CT interactions. Thirdly, the preparation of macrocyclic molecules should be easy, and the raw materials should be cheap and easy to obtain. Adamantane and its derivatives, which have a highly symmetrical and rigid structure, can pack tightly with each other to form a lattice, and thus can easily form crystals in organic solvents; they have been widely used in the field of crystal engineering [44,45,46,47,48,49,50,51]. Building blocks 1,3-Dimethoxybenzene, that can act as π-donors, are frequently used in the construction of supramolecular macrocyclic molecules [37,52]. We reasoned that new macrocyclic molecules, made by combining easily crystallized adamantane groups with π-donor 1,3-dimethoxy-benzene units, might have the potential for the development of new macrocyclic molecule-based CT cocrystals with π-acceptor guests.

**Scheme 1:**
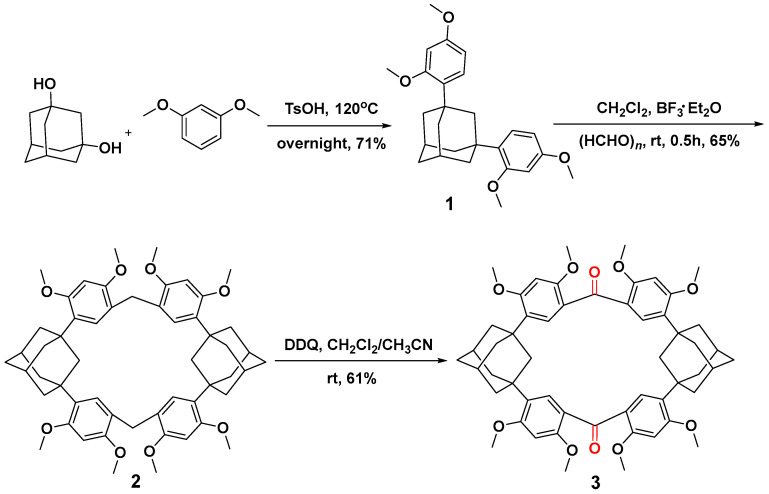
The synthesis route of macrocyclic molecules **2** and **3**.

**Figure 1:**
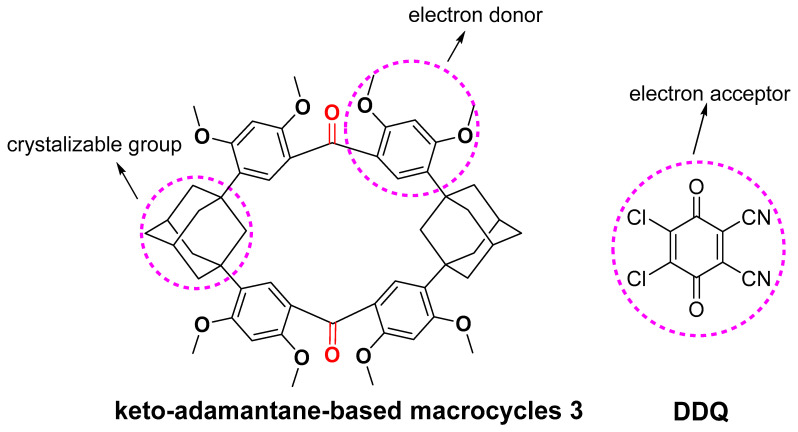
Chemical structures of **3** and DDQ.

Herein, we report the synthesis of the adamantane-based macrocycles **3** and **2** (Scheme 1). Molecule **2** can be easily prepared by the reaction of 1,3-bis(2,4-dimethoxyphenyl)adamantane and paraformaldehyde, in the presence of boron trifluoride-diethyl etherate as the catalyst. The oxidation of **2** using 2,3-dichloro-5,6-dicyano-1,4-benzoquinone (DDQ) as an oxidant can afford a keto-adamantane-based macrocycle **3**. Further studies demonstrated the formation of a new type of macrocyclic molecule-based CT cocrystal, through exo-wall CT interactions between **3** and DDQ (Figure 1). This CT co-crystal can be produced during the simple rotary evaporation of the dichloromethane solution of **3** and DDQ, rather than by slow crystallization. The cocrystal material showed a selective vapochromism behavior towards THF, specifically, among nine volatile organic solvents commonly used in the laboratory. Our research provides a new strategy for the design and synthesis of macrocyclic molecule-based CT cocrystals that can show selective vapochromic properties towards organic solvent molecules.

## 2. Results and Discussion

The synthesis of **3** is shown in Scheme 1. Starting from commercially available 1,3-dimethoxy-benzene and 1,3-dihydroxyadamantane, 1,3-bis(2,4-dimethoxyphenyl)adamantine **1** can be obtained in 71% yield through the Friedel–Crafts reaction. Combining **1** and paraformaldehyde in anhydrous dichloromethane at room temperature in the presence of borontrifluoride diethyl etherate as the catalyst, adamantane-based macrocycle **2** was obtained with 65% yield. With macrocycle **2** in hand, we attempted to use **2** and DDQ to construct macrocycle **2**-based CT cocrystal materials. To our surprise, after the mixture of **2** and DDQ in a 1:1 molar ratio in CH_2_Cl_2_, a large amount of purple-black precipitate was formed immediately. Further research results including ^1^H NMR and ^13^CNMR, indicated that this purple-black precipitate was the macrocyclic molecule **3**, which was the product of the oxidation of the bridged methylene of **2**. After optimization of the reaction conditions, **3** could be produced with 61% yield in the presence of 10.0 equiv. DDQ in CH_2_Cl_2_/CH_3_CN (*v*/*v* = 1:1).

**Figure 2:**
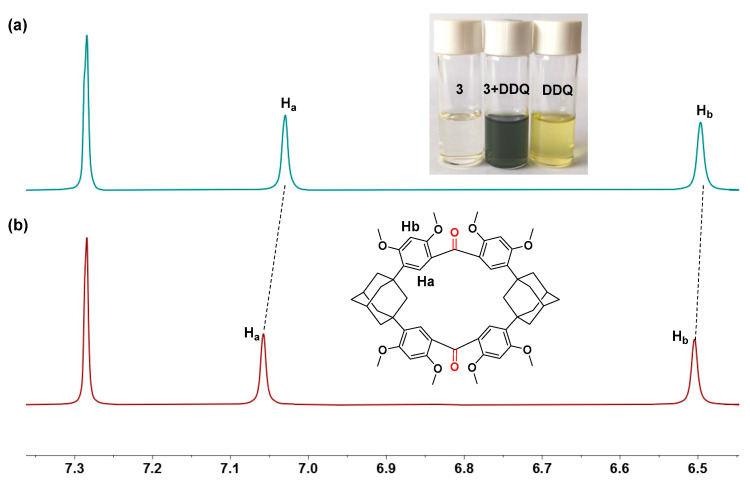
Partial ^1^H NMR spectra (400 MHz, CDCl_3_, 298 K) of (**a**) **3** and 1.0 equiv. of DDQ, and (**b**) free **3**. [3]_0_ = 4.0 mM. Inset: photograph showing colors of **3**, **3** + DDQ and DDQ in CHCl_3_.

Considering the presence of electron-rich 1,3-dimethoxybenzene groups, we assumed that **3** might form crystalline CT assemblies with DDQ, so the interactions between **3** and DDQ were investigated in CDCl_3_. After the addition of **3** to DDQ in a 1:1 molar ratio in CHCl_3_, an obvious color change from yellow to dark green was observed, indicating the interaction between **3** and DDQ occurred (Figure 2, inset). The absorption measurements of ^1^H NMR and UV-Vis further confirmed the interaction between **3** and DDQ in CDCl_3_ solutions. As shown in Figure 2, when **3** (4.0 mM) and 1.0 equiv. DDQ were mixed in CDCl_3_, a new set of signals that were different from those of **3** were observed, suggesting the formation of a **3·**DDQ complex. The complexation and decomplexation between **3** and DDQ were too fast to differentiate using the NMR technique at room temperature. The protons H_a_ and H_b_ that correspond to **3**, shifted upfield by 0.03 and 0.01 ppm, respectively. Moreover, a strong new absorption peak appeared at 319 nm for the CDCl_3_ solution of **3** and DDQ (Figure S7). All these results revealed the formation of a CT complex between **3** and DDQ.

**Figure 3:**
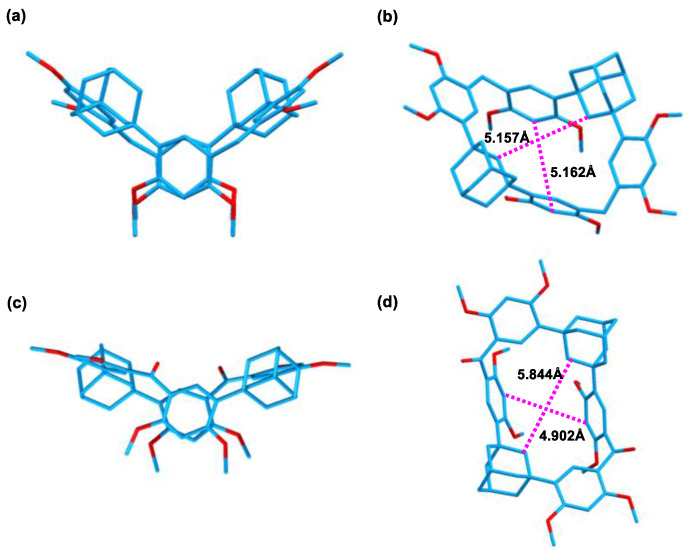
Single crystal structures of **2** (**a**,**b**); single crystal structures of **3** (**c**,**d**).

Single crystals of **2** and **3** that are suitable for X-ray analysis were obtained by slow evaporation of the dichloromethane solution of **2** (CCDC: 2258292) and **3** (CCDC: 2258293). As shown in Figure 3, the macrocyclic molecule **2** adopts a cone configuration. The distance between the two C atoms of adamantane residue is measured to be 5.157 Å, indicating the small size of the cavity of **2**. Similar to **2**, keto-adamantane-based macrocyclic molecule **3** also chose a cone configuration in the solid state. The distance between the two C atoms of adamantane residue is measured to be 5.844 Å, which is slightly longer than that of **2**. However, the distance between the two C atoms of 1,3-dimethoxy-benzene units is measured to be 4.902 Å. This distance value is shorter than that of 5.162 Å of **2**, suggesting the cavity of **3** is too small to accommodate DDQ with sizes of 5.231 Å × 6.206 Å. Therefore, we can infer that the complex behavior of **3** and DDQ in CDCl_3_ occurred through the exo-wall CT interactions between the 1,3-dimethoxy-benzene units of **3** and DDQ.

**Figure 4:**
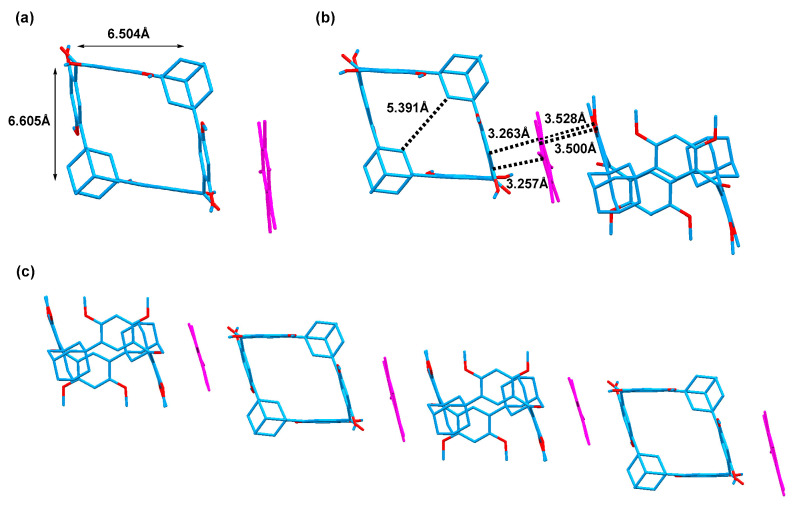
Single crystal structures of **3**@DDQ (**a**,**b**); stacking mode of **3**@DDQ (**c**).

A dark green single crystal of **3**@DDQ (CCDC: 2258294) that was suitable for X-ray analysis was grown by slow evaporation of the dichloromethane solution of **3** and DDQ in 1:1 molar ratio. As shown in Figure 4, after complexation with DDQ, **3** exhibited a box-like structure in the solid state with a size of 6.504 Å × 6.605 Å, and the 1,3-dimethoxy-benzene rings became parallel to the opposite one. The distance between these two C atoms of adamantane residue was measured to be 5.391 Å. The 1,3-dimethoxy-benzenerings of **3** and DDQ were orientated in a face-to-face manner and showed π···π stacking interactions with distances of 3.263 Å, 3.257 Å, 3.528 Å and 3.500 Å (f), which were indicative of strong CT interactions between these π-systems. Because of these multiple π…π stacking interactions, a linear supramolecular array between **3** and DDQ was further formed in the solid state. All these results suggested the formation of a novel macrocyclic molecule **3**-based CT co-crystal.

**Figure 5:**
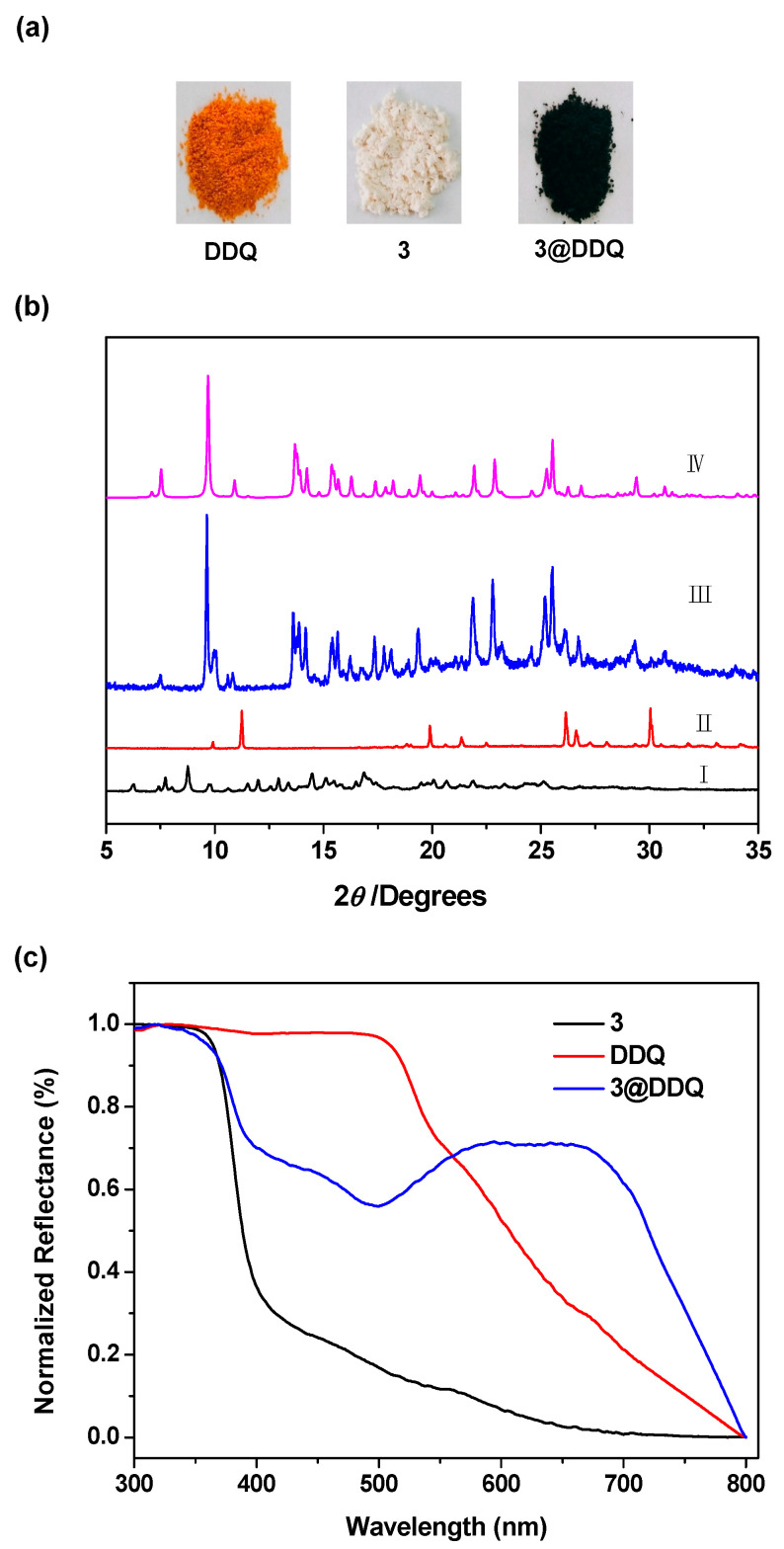
Photographs of **3**, **3**@DDQ and DDQ (**a**); PXRD patterns of **3**, **3**@DDQ and DDQ (**b**). I: **3**, II: DDQ, III: **3**@DDQ, IV: simulated from **3**@DDQ; (**c**) diffuse reflectance spectra of **3**, **3**@DDQ and DDQ.

We assumed that the co-crystal materials might be prepared by evaporation of the solvent. Consequently, co-crystals **3**@DDQ can be prepared in gram scale by evaporation of the CH_2_Cl_2_ solution of **3** and DDQ in 1:1 molar ratio. The ^1^H NMR and TGA results demonstrated the complete removal of CH_2_Cl_2_ (Figures S8 and S9). The color of the as-obtained material is neither the white of **3** nor the yellow of DDQ, but the same dark green color as that of the single crystal **3**@DDQ (Figure 5a). The powder X-ray diffraction (PXRD) pattern of this material shows sharp peaks, indicating that it is a crystalline material. In addition, the PXRD patterns, that are different from **3** or DDQ, are consistent with the simulated patterns of single crystal **3**@DDQ, indicating the formation of **3**@DDQ co-crystal material (Figure 5b). Solid-state UV-Vis diffuse reflectance spectroscopy was used to investigate the formation of **3**@DDQ co-crystal material as well. As shown in Figure 5c, the as-prepared material showed a strong absorption peak in the range of 500–700 nm, which was totally different to those of **3** and DDQ, suggesting the formation of **3**@DDQ co-crystal during evaporation.

**Figure 6:**
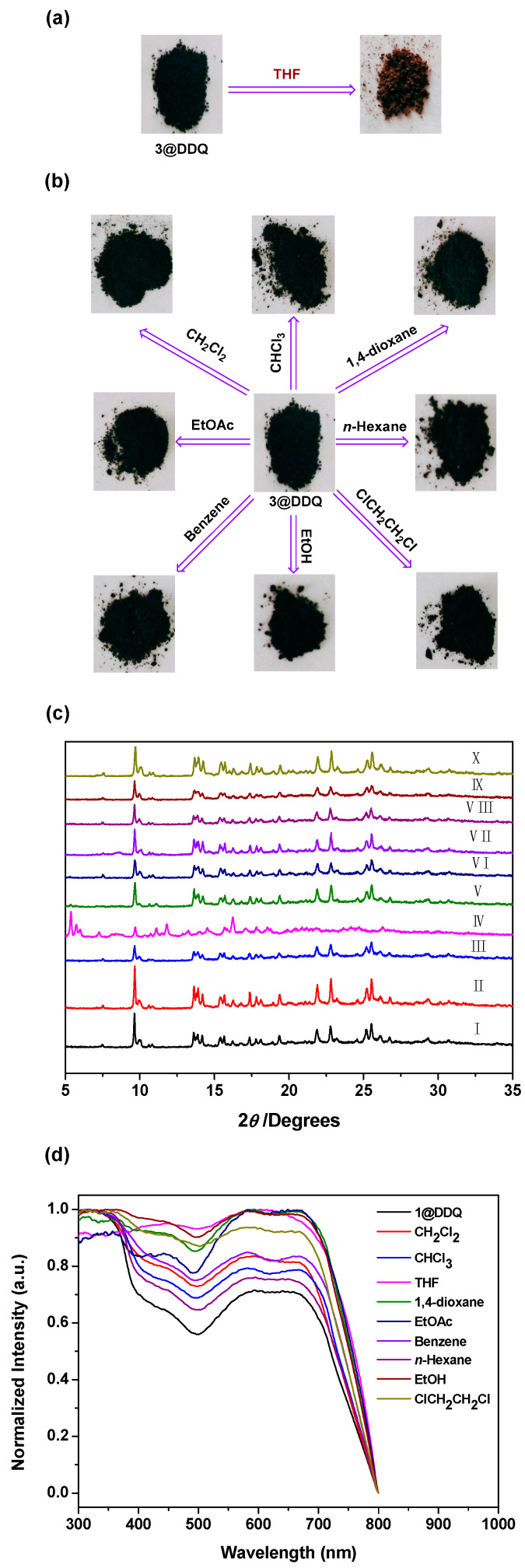
Photographs of **3**@DDQ after exposure to various vapors (**a**,**b**); (**c**) PXRD patterns of **3**@DDQ before (I) and after (II) exposure to CH_2_Cl_2_, (III) CHCl_3_, (IV) THF, (V) 1,4-dioxane, (VI) EtOAc, (VII) benzene, (VIII) *n*-hexane, (IX) EtOH and (X) ClCH_2_CH_2_Cl; (**d**) diffuse reflectance spectra of **3**@DDQ before and after exposure to various vapors.

In order to investigate the properties of **3**@DDQ, the vapochromic behavior of **3**@DDQ was studied. Organic solvents that are commonly used in laboratories were chosen because these solvent vapors are difficult to distinguish without the assistance of instrumental analysis. Our research suggested that **3**@DDQ showed a selective vapochromic response to THF vapor. As shown in Figure 6a, the color of **3**@DDQ changed from dark green to brown after 12 h when it was exposed to THF vapor. Additionally, after the samples were removed from the THF vapor and left at room temperature for 24 h, the color changed from brown to dark green. However, no color change was observed when **3**@DDQ was exposed to other vapors, including CH_2_Cl_2_; CHCl_3_; 1,4-dioxane; EtOAc; benzene; *n*-hexane; EtOH; and ClCH_2_CH_2_Cl, at room temperature (Figure 6b). It should be noted that CH_2_Cl_2_, CHCl_3_, 1,4-dioxane, benzene, and THF can be adsorbed by **3**@DDQ, while only THF can trigger the crystal-to-crystal transformation. These results indicate the selectivity of the vapochromic properties of **3**@DDQ. To further investigate the mechanism of the THF vapor-induced color change of **3**@DDQ, PXRD experiments were carried out. After exposure to THF vapor, obvious changes of the PXRD patterns of **3**@DDQ were observed. New sharp peaks at 5.37, 5.77, 11.12 and 11.85 degrees were observed, confirming the transformation of **3**@DDQ after the adsorption of THF (Figure 6c). This transformation induced a color change. After the exposure of **3**@DDQ to other vapors, the PXRD patterns of **3**@DDQ did not change, indicating no crystal-to-crystal transformation occurred for **3**@DDQ. We tried to grow the single crystal of **3**@DDQ by slow evaporation of the solution of **3** and 1.0 equiv. DDQ in THF, but only white and yellow powders instead of dark green crystals were obtained. These results indicate that the transformation of **3**@DDQ crystals after the adsorbance of THF vapors may be due to the decomplexation of **3**@DDQ. Solid-state UV-Vis diffuse reflectance spectroscopy experiments were used to study the vapochromic behavior of **3**@DDQ. After the adsorption of THF molecules, the absorbance of **3**@DDQ at~500 nm became significantly enhanced compared with other vapors, indicating the decomplexation of **3**@DDQ and the release of DDQ (Figure 6d). The results of ^1^H NMR further support the decomplexation of **3**@DDQ upon its exposure to THF vapor. When **3** (4.0 mM) and 1.0 equiv. DDQ were mixed in THF-*d8*, the proton signals of **3** did not shift, indicating no **3**·DDQ complex was formed. This is consistent with the results showing that THF can trigger the decomplexation of **3**·DDQ (Figure S28).

## 3. Materials and Methods

### 3.1. General Considerations

All reactions were carried out with oven-dried glassware. Commercial reagents were used without further purification. Flash column chromatography was performed on 100–200 mesh silica gel. The spectra of ^1^H NMR and ^13^C NMR were recorded on a Bruker DMX400 NMR spectrometer. Melting points were determined using WRR melting point apparatus and were uncorrected. High-resolution atmospheric pressure chemical ionization mass spectra (APCI-MS) were determined by Bruker Daltonics. Inc., Billerica, MA, USA.

Crystal structure determinationSingle-crystal data for **2**, **3** and **3**@DDQ were collected using a Bruker Smart Apex II diffractometer (Bruker, Germany) with Mo-Kα radiation (λ = 0.71073 Å). All empirical absorption corrections were applied using the SADABS program [53]. All structures were solved using direct methods, which yielded the positions of all non-hydrogen atoms. The positions were first refined isotropically, then anisotropically. All hydrogen atoms of the ligands were placed in calculated positions with fixed isotropic thermal parameters, and were included in the structure factor calculations in the final stage of full-matrix least-squares refinement. All calculations were performed using the SHELXTL 5.1 software package [54]. 

Powder X-ray diffraction (PXRD) data were collected on a Rigaku Ultimate-IV X-ray diffractometer operating at 40 kV/30 mA using the Cu K*α* line (*λ* = 1.5418 Å). Data were measured over the range of 5–45° in 5°/min steps over 8 min.

Thermogravimetric analysis (TGA) was carried out using a Q5000IR analyzer (TA Instruments) with an automated vertical overhead thermobalance. The samples were heated at 10 °C/min using N_2_ as the protective gas.

Vapochromic experiments. An open 2 mL vial containing 10 mg of **3**@DDQ was placed in a sealed 20 mL vial containing 1 mL of each vapor solution. **3**@DDQ powders were exposed under saturated vapor pressure in the closed vessel at room temperature. Obvious color changes were observed after 12 h. 

Sample preparation of **3**@DDQ. DDQ (282 mg) and **3** (1085 mg) and were dissolved in 100 mL CH_2_Cl_2_, and then rapid evaporation of the solution under vacuum at 50 °C afforded dark green cocrystals of **3**@DDQ.

### 3.2. Typical Procedure for Synthesis of 1, 2, 3

Compound **1**. A mixture of 1,3-dihydroxyadamantane (1680 mg, 10 mmol), 1,3-dimethoxy-benzene (9660 mg, 70 mmol), and TsOH (860 mg, 5 mmol) in a flask was stirred at 120 °C overnight under N_2_. After quenching the reaction with water (100 mL), the resulting mixture was extracted with dichloromethane (3 × 50 mL) and then washed with water and brine successively. The organic layer was dried over anhydrous Na_2_SO_4_ and evaporated. The residue was purified using column chromatography with dichloromethane/petroleum ether as eluent, to afford compound **1** (2900 mg, yield 71%) as a white solid. ^1^H NMR (400 MHz, CDCl_3_) δ 7.19 (d, *J* = 8.4 Hz, 2H), 6.53–6.43 (m, 4H), 3.82 (s, 12H), 2.40 (d, *J* = 10.6 Hz, 2H), 2.25–2.02 (m, 10H), 1.79 (s, 2H). ^13^C NMR (101 MHz, CDCl_3_) δ 159.7, 158.8, 131.2, 126.9, 103.4, 99.7, 55.3, 55.0, 43.6, 40.6, 40.4, 37.7, 37.4, 36.7, 29.9, 29.9. HRMS (APCI) *m*/*z*: [M + H]^+^ calcd for C_26_H_33_O_4_, 409.2379; found, 409.2385. Anal. calcd for C_26_H_32_O_4_: C, 76.44; H, 7.90. Found: C, 76.41; H, 7.92.

Compound **2**. To a mixture of **1** (820 mg, 2.0 mmol) and paraformaldehyde (180 mg, 6.0 mmol) in dichloromethane (150 mL), a catalytic amount of boron trifluoride diethyl etherate (0.3 mL, 2.4 mmol) was added. The mixture was stirred at room temperature for 0.5 h. Then the reaction was quenched by the addition of 150 mL water. The organic layer was separated and dried with anhydrous MgSO_4_. The solvent was removed in vacuo and the residue was separated using column chromatography on silica gel (eluent: 3:1 DCM/Petroleum ether) to give **2** (546 mg, 65%) as yellow solids. ^1^H NMR (400 MHz, CDCl_3_) δ 6.49 (s, 4H), 6.42 (s, 4H), 3.75 (d, *J* = 3.4 Hz, 28H), 2.13–1.96 (m, 12H), 1.80–1.57 (m, 16H). ^13^C NMR (101 MHz, CDCl_3_) δ 157.8, 156.2, 130.5, 127.9, 120.1, 96.4, 55.6, 55.5, 44.8, 40.1, 37.1, 36.2, 29.7, 28.5. HRMS (APCI) *m*/*z*: [M + H]^+^ calcd for C_54_H_65_O_8_, 841.4679; found, 841.4675. Anal. calcd for C_54_H_64_O_8_: C, 77.11; H, 7.67. Found: C, 77.09; H, 7.68.

Compound **3**. A mixture of **2** (4200 mg, 5 mmol) and DDQ (11.3 × 10^3^ mg, 50 mmol) in CH_2_C_l2_/CH_3_CN (*v*/*v* = 1:1) was stirred at room temperature overnight. The mixture was evaporated and purified using column chromatography with dichloromethane/CH_3_OH as eluent to afford compound **3** (2650 mg, yield 61%) as a white solid. ^1^H NMR (400 MHz, CDCl_3_) δ 7.06 (s, 4H), 6.50 (s, 4H), 3.90 (d, *J* = 16.5 Hz, 24H), 2.10 (s, 6H), 1.70 (s, 6H), 1.59–1.53 (m, 16H). ^13^C NMR (101 MHz, CDCl_3_) δ193.8, 155.0, 129.1, 97.0, 56.5, 55.2, 45.2, 36.7, 36.0, 29.4. HRMS (APCI) *m*/*z*: [M + H]^+^ calcd for C_54_H_61_O_10_, 869.4265; found, 869.4271. Anal. calcd for C_54_H_60_O_10_: C, 74.63; H, 6.96. Found: C, 74.64; H, 6.98.

## 4. Conclusions

In summary, we have successfully constructed a new macrocyclic molecule **2** by combining adamantane building blocks with π-donor 1,3-dimethoxy-benzene units. The oxidation of **2** using DDQ as an oxidant resulted in the formation of a keto-adamantane-based macrocycle **3** in high yield. Interestingly, **3** can form CT co-crystal 1@DDQ by evaporation of a solution of **3** and DDQ in CH_2_Cl_2_ in a 1:1 molar ratio. A selective vapochromic response to THF vapor, specifically, among nine commonly used organic solvent vapors, was shown by **3**@DDQ. The single crystal structure of **3**@DDQ indicated that **3**@DDQ was formed through the exo-wall CT interactions of **3** and DDQ. PXRD curves indicate that the mechanism of vapochromism comes from the THF-induced decomplexation of **3**@DDQ. The advantages of easy preparation, selective vapochromism behavior towards THF vapor, and definite assembly structure endow CT co-crystal **3**@DDQ with enormous potential for application in gas detection, adsorption, and separation. The fabrication of other CT co-crystals using **3** and other guests, and their possible applications, are ongoing in our laboratory.

## Data Availability

Data are contained within the article and supplementary materials.

## Supplementary Materials

The following supporting information can be downloaded at https://www.mdpi.com/article/10.3390/molecules29030719/s1, ^1^H, ^13^C spectra of the new compounds; Uv-vis spectral of **3** and DDQ; Characterization of **3**@DDQ; vapor adsorption experiments; complexation between **3** and DDQ in THF; crystal data.
