# Supplementary material for: Keto-Adamantane-Based Macrocycle Crystalline Supramolecular Assemblies Showing Selective Vapochromism to Tetrahydrofuran
Source: Molecules. 2024 Feb 4;29(3):719. doi: 10.3390/molecules29030719 (PMC10856198; doi:10.3390/molecules29030719)

checkCIF/PLATON report

You have not supplied any structure factors. As a result the full set of tests cannot be run.

THIS REPORT IS FOR GUIDANCE ONLY. IF USED AS PART OF A REVIEW PROCEDURE FOR PUBLICATION, IT SHOULD NOT REPLACE THE EXPERTISE OF AN EXPERIENCED CRYSTALLOGRAPHIC REFEREE.

No syntax errors found. CIF dictionary Interpreting this report

Datablock: 11

Bond precision:	C-C = 0.0038 A		Wavelength=0.71073
Cell:	a=18.227(5)	b=18.218(5)	c=19.010(5)
	alpha=90	beta=111.022(4)	gamma=90
Temperature:	296 K		

	Calculated	Reported
Volume	5892(3)	5892(3)
Space group	C 2/c	C 1 2/c 1
Hall group	-C 2yc	-C 2yc
Moiety formula	2(C54 H60 O10), C16 Cl4 N4 O4, 2(C H2 Cl), 2(Cl)	2(C54 H60 O10), C2 H4 Cl4, C16 Cl4 N4 O4
Sum formula	C126 H124 Cl8 N4 O24	C126 H124 Cl8 N4 O24
Mr	2361.89	2361.88
Dx,g cm-3	1.331	1.331
Z	2	2
Mu (mm-1)	0.265	0.265
F000	2472.0	2472.0
F000’	2475.49	
h,k,lmax	23,23,24	23,23,24
Nref	6793	6693
Tmin,Tmax		0.703,0.746
Tmin’		

Correction method= # Reported T Limits: Tmin=0.703 Tmax=0.746
AbsCorr = NONE

Data completeness= 0.985 Theta(max)= 27.527

R(reflections)= 0.0801(4222) wR2(reflections)= 0.2671(6693)

S = 1.041 Npar= 434

The following ALERTS were generated. Each ALERT has the format
test-name_ALERT_alert-type_alert-level.
Click on the hyperlinks for more details of the test.

Alert level C

```
PLAT042_ALERT_1_C Calc. and Reported MoietyFormula Strings  Differ     Please Check
PLAT053_ALERT_1_C Minimum Crystal Dimension Missing (or Error) ...     Please Check
PLAT054_ALERT_1_C Medium  Crystal Dimension Missing (or Error) ...     Please Check
PLAT055_ALERT_1_C Maximum Crystal Dimension Missing (or Error) ...     Please Check
PLAT084_ALERT_3_C High wR2 Value (i.e. > 0.25) ..................        0.27 Report
PLAT094_ALERT_2_C Ratio of Maximum / Minimum Residual Density ....       2.19 Report
PLAT260_ALERT_2_C Large Average Ueq of Residue Including       Cl1      0.155 Check
PLAT260_ALERT_2_C Large Average Ueq of Residue Including        Cl      0.176 Check
PLAT329_ALERT_4_C Carbon Atom Hybridisation Unclear for .........          C1 Check
```

Alert level G

```
PLAT002_ALERT_2_G Number of Distance or Angle Restraints on AtSite          1 Note
PLAT003_ALERT_2_G Number of Uiso or Uij Restrained non-H Atoms ...         14 Report
PLAT012_ALERT_1_G No       _shelx_res_checksum Found in CIF ......     Please Check
PLAT072_ALERT_2_G SHELXL First  Parameter in WGHT  Unusually Large       0.17 Report
PLAT176_ALERT_4_G The CIF-Embedded .res File Contains SADI Records          1 Report
PLAT178_ALERT_4_G The CIF-Embedded .res File Contains SIMU Records          1 Report
PLAT188_ALERT_3_G A Non-default SIMU Restraint Value has been used     0.0150 Report
PLAT300_ALERT_4_G Atom Site Occupancy of Cl2        Constrained at        0.5 Check
PLAT300_ALERT_4_G Atom Site Occupancy of Cl3A       Constrained at        0.5 Check
PLAT300_ALERT_4_G Atom Site Occupancy of O1         Constrained at        0.5 Check
PLAT300_ALERT_4_G Atom Site Occupancy of O2A        Constrained at        0.5 Check
PLAT300_ALERT_4_G Atom Site Occupancy of N1         Constrained at        0.5 Check
PLAT300_ALERT_4_G Atom Site Occupancy of N1A        Constrained at        0.5 Check
PLAT300_ALERT_4_G Atom Site Occupancy of C2         Constrained at        0.5 Check
PLAT300_ALERT_4_G Atom Site Occupancy of C2A        Constrained at        0.5 Check
PLAT300_ALERT_4_G Atom Site Occupancy of C3         Constrained at        0.5 Check
PLAT300_ALERT_4_G Atom Site Occupancy of C3A        Constrained at        0.5 Check
PLAT300_ALERT_4_G Atom Site Occupancy of C4         Constrained at        0.5 Check
PLAT300_ALERT_4_G Atom Site Occupancy of C4A        Constrained at        0.5 Check
PLAT300_ALERT_4_G Atom Site Occupancy of C5         Constrained at        0.5 Check
PLAT300_ALERT_4_G Atom Site Occupancy of C6A        Constrained at        0.5 Check
PLAT300_ALERT_4_G Atom Site Occupancy of Cl1        Constrained at        0.5 Check
PLAT300_ALERT_4_G Atom Site Occupancy of C1         Constrained at        0.5 Check
PLAT300_ALERT_4_G Atom Site Occupancy of H1A        Constrained at        0.5 Check
PLAT300_ALERT_4_G Atom Site Occupancy of H1B        Constrained at        0.5 Check
PLAT300_ALERT_4_G Atom Site Occupancy of Cl         Constrained at        0.5 Check
PLAT302_ALERT_4_G Anion/Solvent/Minor-Residue Disorder (Resd  2  )       100% Note
PLAT302_ALERT_4_G Anion/Solvent/Minor-Residue Disorder (Resd  3  )       100% Note
PLAT302_ALERT_4_G Anion/Solvent/Minor-Residue Disorder (Resd  4  )       100% Note
PLAT304_ALERT_4_G Non-Integer Number of Atoms in ..... (Resd  4  )       0.50 Check
PLAT432_ALERT_2_G Short Inter X...Y Contact  Cl       ..C1       .       1.56 Ang.
                                     3/2-x,1/2-y,1-z  =      7_656 Check
PLAT432_ALERT_2_G Short Inter X...Y Contact  O005     ..C6A      .       2.90 Ang.
                                         1-x,y,3/2-z  =      2_656 Check
PLAT432_ALERT_2_G Short Inter X...Y Contact  O005     ..C4A      .       2.99 Ang.
                                               x,y,z  =      1_555 Check
PLAT432_ALERT_2_G Short Inter X...Y Contact  O005     ..C6A      .       3.00 Ang.
                                               x,y,z  =      1_555 Check
```

```
PLAT720_ALERT_4_G Number of Unusual/Non-Standard Labels ..........         61 Note
PLAT764_ALERT_4_G Overcomplete CIF Bond List Detected (Rep/Expd) .       1.27 Ratio
PLAT773_ALERT_2_G Check long C-C Bond in CIF: C3       --C4            1.98 Ang.
PLAT773_ALERT_2_G Check long C-C Bond in CIF: C3       --C4A           1.78 Ang.
PLAT773_ALERT_2_G Check long C-C Bond in CIF: C2A      --C4A           1.96 Ang.
PLAT773_ALERT_2_G Check long C-C Bond in CIF: C4A      --C6A           1.79 Ang.
PLAT779_ALERT_4_G Suspect or Irrelevant (Bond) Angle(s) in CIF ...      26.30 Deg.
              C4   -C3   -C4      1_555   1_555   2_656 ......     #  134 Check
PLAT779_ALERT_4_G Suspect or Irrelevant (Bond) Angle(s) in CIF ...      36.20 Deg.
              C3A  -C3   -C4A     2_656   1_555   1_555 ......     #  141 Check
PLAT779_ALERT_4_G Suspect or Irrelevant (Bond) Angle(s) in CIF ...      16.00 Deg.
              C4A  -C3   -C4A     1_555   1_555   2_656 ......     #  145 Check
PLAT779_ALERT_4_G Suspect or Irrelevant (Bond) Angle(s) in CIF ...      27.60 Deg.
              C2   -C4   -O1      1_555   1_555   2_656 ......     #  153 Check
PLAT779_ALERT_4_G Suspect or Irrelevant (Bond) Angle(s) in CIF ...      10.70 Deg.
              O1   -C4   -CL2     1_555   1_555   2_656 ......     #  157 Check
PLAT779_ALERT_4_G Suspect or Irrelevant (Bond) Angle(s) in CIF ...      29.50 Deg.
              C3   -C2   -C2A     2_656   1_555   1_555 ......     #  160 Check
PLAT779_ALERT_4_G Suspect or Irrelevant (Bond) Angle(s) in CIF ...      33.40 Deg.
              C4   -C2   -C4      2_656   1_555   1_555 ......     #  163 Check
PLAT779_ALERT_4_G Suspect or Irrelevant (Bond) Angle(s) in CIF ...      22.80 Deg.
              O1   -C2   -CL2     2_656   1_555   1_555 ......     #  173 Check
PLAT779_ALERT_4_G Suspect or Irrelevant (Bond) Angle(s) in CIF ...      20.60 Deg.
              CL3A -C2A  -N1      1_555   1_555   2_656 ......     #  175 Check
PLAT779_ALERT_4_G Suspect or Irrelevant (Bond) Angle(s) in CIF ...      29.20 Deg.
              C3   -C2A  -C4A     2_656   1_555   2_656 ......     #  180 Check
PLAT779_ALERT_4_G Suspect or Irrelevant (Bond) Angle(s) in CIF ...      19.40 Deg.
              C2   -C2A  -C4      1_555   1_555   2_656 ......     #  187 Check
PLAT779_ALERT_4_G Suspect or Irrelevant (Bond) Angle(s) in CIF ...      20.70 Deg.
              C3A  -C2A  -C4A     1_555   1_555   2_656 ......     #  193 Check
PLAT779_ALERT_4_G Suspect or Irrelevant (Bond) Angle(s) in CIF ...      26.30 Deg.
              C5   -C2A  -CL3A    2_656   1_555   1_555 ......     #  195 Check
PLAT779_ALERT_4_G Suspect or Irrelevant (Bond) Angle(s) in CIF ...       6.40 Deg.
              C5   -C2A  -N1      2_656   1_555   2_656 ......     #  200 Check
PLAT779_ALERT_4_G Suspect or Irrelevant (Bond) Angle(s) in CIF ...      39.90 Deg.
              O2A  -C3A  -C6A     1_555   1_555   2_656 ......     #  204 Check
PLAT779_ALERT_4_G Suspect or Irrelevant (Bond) Angle(s) in CIF ...      28.90 Deg.
              C2A  -C3A  -C5      1_555   1_555   2_656 ......     #  206 Check
PLAT779_ALERT_4_G Suspect or Irrelevant (Bond) Angle(s) in CIF ...       8.70 Deg.
              C4A  -C3A  -C4A     2_656   1_555   1_555 ......     #  210 Check
PLAT779_ALERT_4_G Suspect or Irrelevant (Bond) Angle(s) in CIF ...      30.70 Deg.
              C6A  -C4A  -O2A     1_555   1_555   2_656 ......     #  225 Check
PLAT779_ALERT_4_G Suspect or Irrelevant (Bond) Angle(s) in CIF ...      35.60 Deg.
              N1   -C5   -CL3A    1_555   1_555   2_656 ......     #  231 Check
PLAT779_ALERT_4_G Suspect or Irrelevant (Bond) Angle(s) in CIF ...      44.40 Deg.
              C6A  -N1A  -O2A     1_555   1_555   2_656 ......     #  233 Check
PLAT779_ALERT_4_G Suspect or Irrelevant (Bond) Angle(s) in CIF ...      24.40 Deg.
              C4   -O1   -C4      1_555   1_555   2_656 ......     #  234 Check
PLAT793_ALERT_4_G Model has Chirality at C00R        (Centro SPGR)          S Verify
PLAT793_ALERT_4_G Model has Chirality at C00U        (Centro SPGR)          R Verify
PLAT860_ALERT_3_G Number of Least-Squares Restraints ............         85 Note
PLAT933_ALERT_2_G Number of HKL-OMIT Records in Embedded .res File         12 Note
```

---

0 **ALERT level A** = Most likely a serious problem - resolve or explain
0 **ALERT level B** = A potentially serious problem, consider carefully
9 **ALERT level C** = Check. Ensure it is not caused by an omission or oversight

```
65 ALERT level G = General information/check it is not something unexpected

 5 ALERT type 1 CIF construction/syntax error, inconsistent or missing data
15 ALERT type 2 Indicator that the structure model may be wrong or deficient
 3 ALERT type 3 Indicator that the structure quality may be low
51 ALERT type 4 Improvement, methodology, query or suggestion
 0 ALERT type 5 Informative message, check
```

It is advisable to attempt to resolve as many as possible of the alerts in all categories. Often the minor alerts point to easily fixed oversights, errors and omissions in your CIF or refinement strategy, so attention to these fine details can be worthwhile. In order to resolve some of the more serious problems it may be necessary to carry out additional measurements or structure refinements. However, the purpose of your study may justify the reported deviations and the more serious of these should normally be commented upon in the discussion or experimental section of a paper or in the "special_details" fields of the CIF. checkCIF was carefully designed to identify outliers and unusual parameters, but every test has its limitations and alerts that are not important in a particular case may appear. Conversely, the absence of alerts does not guarantee there are no aspects of the results needing attention. It is up to the individual to critically assess their own results and, if necessary, seek expert advice.

Publication of your CIF in IUCr journals

A basic structural check has been run on your CIF. These basic checks will be run on all CIFs submitted for publication in IUCr journals (*Acta Crystallographica*, *Journal of Applied Crystallography*, *Journal of Synchrotron Radiation*); however, if you intend to submit to *Acta Crystallographica Section C* or *E* or *IUCrData*, you should make sure that full publication checks are run on the final version of your CIF prior to submission.

Publication of your CIF in other journals

Please refer to the *Notes for Authors* of the relevant journal for any special instructions relating to CIF submission.

PLATON version of 28/11/2022; check.def file version of 28/11/2022

Datablock 11 - ellipsoid plot

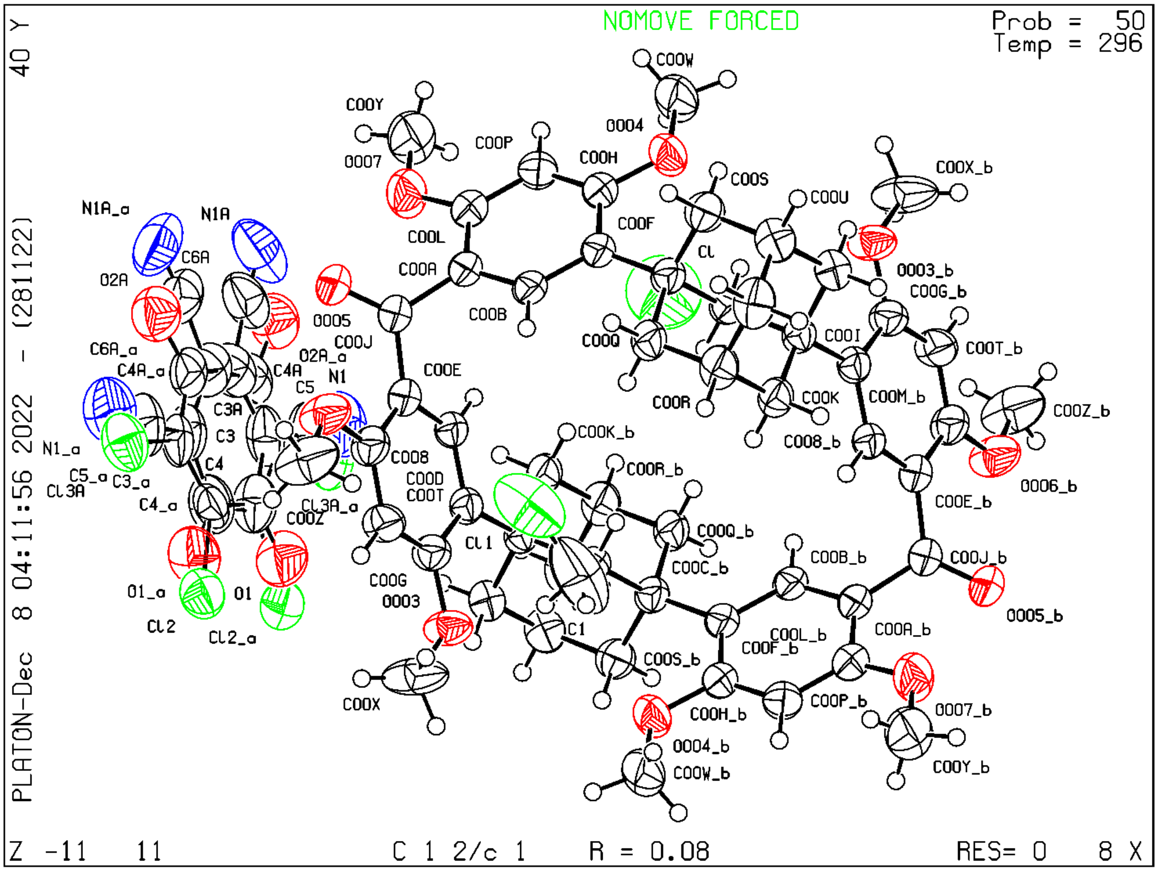

Supplement: Supplementary file 1 [file molecules-29-00719-s001.zip › 11 checkcif.pdf]
