# Supplementary material for: Keto-Adamantane-Based Macrocycle Crystalline Supramolecular Assemblies Showing Selective Vapochromism to Tetrahydrofuran
Source: Molecules. 2024 Feb 4;29(3):719. doi: 10.3390/molecules29030719 (PMC10856198; doi:10.3390/molecules29030719)

checkCIF/PLATON report

Structure factors have been supplied for datablock(s) 11

THIS REPORT IS FOR GUIDANCE ONLY. IF USED AS PART OF A REVIEW PROCEDURE FOR PUBLICATION, IT SHOULD NOT REPLACE THE EXPERTISE OF AN EXPERIENCED CRYSTALLOGRAPHIC REFEREE.

No syntax errors found. CIF dictionary Interpreting this report

Datablock: 11

Bond precision: C-C = 0.0094 A Wavelength=0.71073

Cell:	a=14.54(5)	b=15.25(5)	c=15.42(5)
	alpha=114.04(4)	beta=99.38(4)	gamma=112.59(4)
Temperature:	296 K		

	Calculated	Reported
Volume	2668(15)	2667(15)
Space group	P -1	P -1
Hall group	-P 1	-P 1
Moiety formula	C54 H60 O10, 2(C H2 Cl2) [+ solvent]	C54 H60 O10, 2(C H2 Cl2)
Sum formula	C56 H64 Cl4 O10 [+ solvent]	C56 H64 Cl4 O10
Mr	1038.87	1038.87
Dx,g cm-3	1.293	1.294
Z	2	2
Mu (mm-1)	0.279	0.279
F000	1096.0	1096.0
F000’	1097.67	
h,k,lmax	17,18,18	17,18,18
Nref	9414	9378
Tmin,Tmax	0.946,0.956	0.429,0.746
Tmin’	0.946	

Correction method= # Reported T Limits: Tmin=0.429 Tmax=0.746
AbsCorr = NONE

Data completeness= 0.996 Theta(max)= 25.000

R(reflections)= 0.0908(3441) wR2(reflections)= 0.3195(9378)

S = 0.951 Npar= 639

The following ALERTS were generated. Each ALERT has the format
test-name_ALERT_alert-type_alert-level.
Click on the hyperlinks for more details of the test.

Alert level B

```
PLAT026_ALERT_3_B Ratio Observed / Unique Reflections (too) Low ..        37% Check
```

Alert level C

```
PLAT084_ALERT_3_C High wR2 Value (i.e. > 0.25) ..................       0.32 Report
PLAT148_ALERT_3_C s.u. on the      a    - Axis is (Too) Large ....      0.050 Ang.
PLAT148_ALERT_3_C s.u. on the      b    - Axis is (Too) Large ....      0.050 Ang.
PLAT148_ALERT_3_C s.u. on the      c    - Axis is (Too) Large ....      0.050 Ang.
PLAT234_ALERT_4_C Large Hirshfeld Difference C28      --C33      .       0.16 Ang.
PLAT234_ALERT_4_C Large Hirshfeld Difference C49      --C50      .       0.16 Ang.
PLAT244_ALERT_4_C Low    'Solvent' Ueq as Compared to Neighbors of        C54 Check
PLAT244_ALERT_4_C Low    'Solvent' Ueq as Compared to Neighbors of        C53 Check
PLAT250_ALERT_2_C Large U3/U1 Ratio for Average U(i,j) Tensor ....        2.1 Note
PLAT260_ALERT_2_C Large Average Ueq of Residue Including       Cl1      0.145 Check
PLAT260_ALERT_2_C Large Average Ueq of Residue Including       Cl3      0.180 Check
PLAT340_ALERT_3_C Low Bond Precision on  C-C Bonds ..............    0.00939 Ang.
PLAT905_ALERT_3_C Negative K value in the Analysis of Variance ...    -16.965 Report
PLAT911_ALERT_3_C Missing FCF Refl Between Thmin & STh/L=    0.595         28 Report
```

Alert level G

```
PLAT002_ALERT_2_G Number of Distance or Angle Restraints on AtSite         46 Note
PLAT072_ALERT_2_G SHELXL First  Parameter in WGHT  Unusually Large       0.17 Report
PLAT154_ALERT_1_G The s.u.'s on the Cell Angles are Equal ..(Note)       0.04 Degree
PLAT172_ALERT_4_G The CIF-Embedded .res File Contains DFIX Records          9 Report
PLAT176_ALERT_4_G The CIF-Embedded .res File Contains SADI Records         10 Report
PLAT431_ALERT_2_G Short Inter HL..A Contact  Cl2      ..O6      .        3.14 Ang.
                                                  x,y,z  =      1_555 Check
PLAT605_ALERT_4_G Largest Solvent Accessible VOID in the Structure        114 A**3
PLAT790_ALERT_4_G Centre of Gravity not Within Unit Cell: Resd.  #          2 Note
             C H2 Cl2
PLAT790_ALERT_4_G Centre of Gravity not Within Unit Cell: Resd.  #          3 Note
             C H2 Cl2
PLAT860_ALERT_3_G Number of Least-Squares Restraints ............         256 Note
PLAT941_ALERT_3_G Average HKL Measurement Multiplicity ..........         2.7 Low
PLAT967_ALERT_5_G Note: Two-Theta Cutoff Value in Embedded .res ..       50.0 Degree
PLAT978_ALERT_2_G Number C-C Bonds with Positive Residual Density.          0 Info
```

0 **ALERT level A** = Most likely a serious problem - resolve or explain
1 **ALERT level B** = A potentially serious problem, consider carefully
14 **ALERT level C** = Check. Ensure it is not caused by an omission or oversight
13 **ALERT level G** = General information/check it is not something unexpected

1 ALERT type 1 CIF construction/syntax error, inconsistent or missing data
7 ALERT type 2 Indicator that the structure model may be wrong or deficient
10 ALERT type 3 Indicator that the structure quality may be low
9 ALERT type 4 Improvement, methodology, query or suggestion
1 ALERT type 5 Informative message, check

Validation response form

Please find below a validation response form (VRF) that can be filled in and pasted into your CIF.

```
# start Validation Reply Form
_vrf_PLAT026_11
;
PROBLEM: Ratio Observed / Unique Reflections (too) Low ..        37% Check
RESPONSE: ...
;
_vrf_PLAT084_11
;
PROBLEM: High wR2 Value (i.e. > 0.25) ..................       0.32 Report
RESPONSE: ...
;
_vrf_PLAT148_11
;
PROBLEM: s.u. on the      a    - Axis is (Too) Large ....      0.050 Ang.
RESPONSE: ...
;
_vrf_PLAT234_11
;
PROBLEM: Large Hirshfeld Difference C28      --C33      .       0.16 Ang.
RESPONSE: ...
;
_vrf_PLAT244_11
;
PROBLEM: Low    'Solvent' Ueq as Compared to Neighbors of        C54 Check
RESPONSE: ...
;
_vrf_PLAT250_11
;
PROBLEM: Large U3/U1 Ratio for Average U(i,j) Tensor ....        2.1 Note
RESPONSE: ...
;
_vrf_PLAT260_11
;
PROBLEM: Large Average Ueq of Residue Including       Cl1      0.145 Check
RESPONSE: ...
;
_vrf_PLAT340_11
;
PROBLEM: Low Bond Precision on  C-C Bonds ..............    0.00939 Ang.
RESPONSE: ...
;
_vrf_PLAT905_11
;
PROBLEM: Negative K value in the Analysis of Variance ...    -16.965 Report
RESPONSE: ...
;
_vrf_PLAT911_11
;
PROBLEM: Missing FCF Refl Between Thmin & STh/L=    0.595         28 Report
RESPONSE: ...
;
# end Validation Reply Form
```

It is advisable to attempt to resolve as many as possible of the alerts in all categories. Often the minor alerts point to easily fixed oversights, errors and omissions in your CIF or refinement strategy, so attention to these fine details can be worthwhile. In order to resolve some of the more serious problems it may be necessary to carry out additional measurements or structure refinements. However, the purpose of your study may justify the reported deviations and the more serious of these should normally be commented upon in the discussion or experimental section of a paper or in the "special_details" fields of the CIF. checkCIF was carefully designed to identify outliers and unusual parameters, but every test has its limitations and alerts that are not important in a particular case may appear. Conversely, the absence of alerts does not guarantee there are no aspects of the results needing attention. It is up to the individual to critically assess their own results and, if necessary, seek expert advice.

Publication of your CIF in IUCr journals

A basic structural check has been run on your CIF. These basic checks will be run on all CIFs submitted for publication in IUCr journals (*Acta Crystallographica*, *Journal of Applied Crystallography*, *Journal of Synchrotron Radiation*); however, if you intend to submit to *Acta Crystallographica Section C* or *E* or *IUCrData*, you should make sure that full publication checks are run on the final version of your CIF prior to submission.

Publication of your CIF in other journals

Please refer to the *Notes for Authors* of the relevant journal for any special instructions relating to CIF submission.

PLATON version of 28/11/2022; check.def file version of 28/11/2022

Datablock 11 - ellipsoid plot

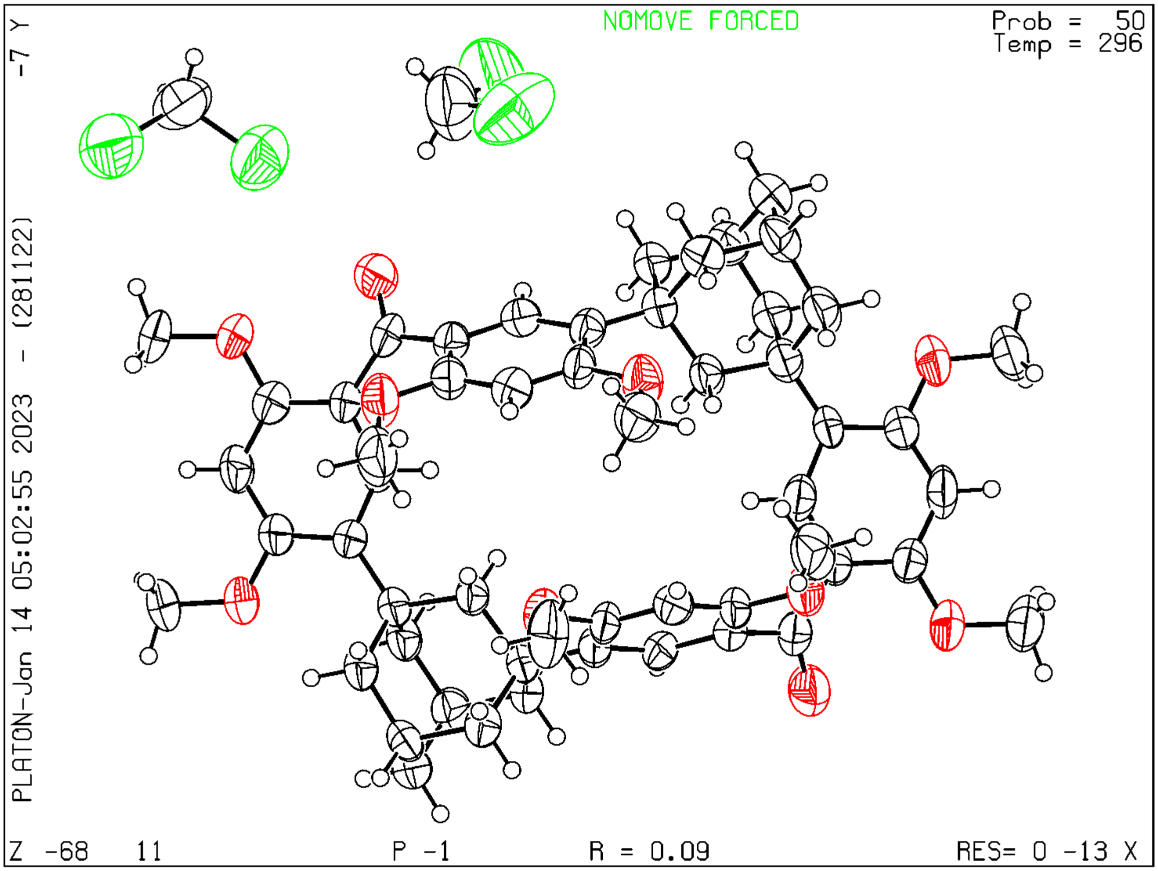

Supplement: Supplementary file 1 [file molecules-29-00719-s001.zip › 111 checkcif.pdf]
