# Supplementary material for: Keto-Adamantane-Based Macrocycle Crystalline Supramolecular Assemblies Showing Selective Vapochromism to Tetrahydrofuran
Source: Molecules. 2024 Feb 4;29(3):719. doi: 10.3390/molecules29030719 (PMC10856198; doi:10.3390/molecules29030719)

checkCIF/PLATON report

Structure factors have been supplied for datablock(s) 2204281_0m

THIS REPORT IS FOR GUIDANCE ONLY. IF USED AS PART OF A REVIEW PROCEDURE FOR PUBLICATION, IT SHOULD NOT REPLACE THE EXPERTISE OF AN EXPERIENCED CRYSTALLOGRAPHIC REFEREE.

No syntax errors found. CIF dictionary Interpreting this report

Datablock: 2204281_0m

Bond precision: C-C = 0.0051 A Wavelength=0.71073

Cell:	a=21.174(3)	b=21.174(3)	c=13.5099(19)
	alpha=90	beta=90	gamma=120
Temperature:	296 K		

	Calculated	Reported
Volume	5245.5(17)	5245.7(17)
Space group	P 31 2 1	P 31 2 1
Hall group	P 31 2"	P 31 2"
Moiety formula	C54 H64 O8 [+ solvent]	C54 H64 O8
Sum formula	C54 H64 O8 [+ solvent]	C54 H64 O8
Mr	841.05	841.05
Dx,g cm-3	0.799	0.799
Z	3	3
Mu (mm-1)	0.053	0.053
F000	1356.0	1356.0
F000'	1356.64	
h,k,lmax	27,27,17	27,27,17
Nref	8052[4347]	7981
Tmin,Tmax	0.994,0.995	0.675,0.746
Tmin'	0.994	

Correction method= # Reported T Limits: Tmin=0.675 Tmax=0.746
AbsCorr = MULTI-SCAN

Data completeness= 1.84/0.99 Theta(max)= 27.481

R(reflections)= 0.0590(4161) wR2(reflections)= 0.1670(7981)

S = 0.974 Npar= 284

The following ALERTS were generated. Each ALERT has the format
test-name_ALERT_alert-type_alert-level.
Click on the hyperlinks for more details of the test.

Alert level C

```
STRVA01_ALERT_4_C          Flack test results are meaningless.
           From the CIF: _refine_ls_abs_structure_Flack    0.000
           From the CIF: _refine_ls_abs_structure_Flack_su    0.600
PLAT213_ALERT_2_C Atom C00V           has ADP max/min Ratio .....        3.2 prolat
PLAT220_ALERT_2_C NonSolvent   Resd 1  C   Ueq(max)/Ueq(min) Range       3.3 Ratio
PLAT222_ALERT_3_C NonSolvent Resd 1  H   Uiso(max)/Uiso(min) Range       4.1 Ratio
PLAT230_ALERT_2_C Hirshfeld Test Diff for    C009     --C00G     .       6.4 s.u.
PLAT230_ALERT_2_C Hirshfeld Test Diff for    C00E     --C00R_a   .       5.7 s.u.
PLAT230_ALERT_2_C Hirshfeld Test Diff for    C00R     --C00E_a   .       5.7 s.u.
PLAT242_ALERT_2_C Low    'MainMol' Ueq as Compared to Neighbors of      O001 Check
PLAT242_ALERT_2_C Low    'MainMol' Ueq as Compared to Neighbors of      O002 Check
PLAT242_ALERT_2_C Low    'MainMol' Ueq as Compared to Neighbors of      O003 Check
PLAT242_ALERT_2_C Low    'MainMol' Ueq as Compared to Neighbors of      O004 Check
PLAT250_ALERT_2_C Large U3/U1 Ratio for Average U(i,j) Tensor ....       2.1 Note
PLAT340_ALERT_3_C Low Bond Precision on  C-C Bonds ...............   0.00514 Ang.
PLAT905_ALERT_3_C Negative K value in the Analysis of Variance ...   -11.836 Report
PLAT905_ALERT_3_C Negative K value in the Analysis of Variance ...    -0.618 Report
PLAT910_ALERT_3_C Missing # of FCF Reflection(s) Below Theta(Min).         5 Note
PLAT911_ALERT_3_C Missing FCF Refl Between Thmin & STh/L=    0.600        10 Report
```

Alert level G

```
PLAT003_ALERT_2_G Number of Uiso or Uij Restrained non-H Atoms ...         6 Report
PLAT032_ALERT_4_G Std. Uncertainty on Flack Parameter Value High .     0.600 Report
PLAT178_ALERT_4_G The CIF-Embedded .res File Contains SIMU Records         1 Report
PLAT186_ALERT_4_G The CIF-Embedded .res File Contains ISOR Records         2 Report
PLAT188_ALERT_3_G A Non-default SIMU Restraint Value has been used    0.0100 Report
PLAT606_ALERT_4_G Solvent Accessible VOID(S) in Structure ........         ! Info
PLAT720_ALERT_4_G Number of Unusual/Non-Standard Labels ..........        62 Note
PLAT791_ALERT_4_G Model has Chirality at C00D         (Sohnke SpGr)        R Verify
PLAT791_ALERT_4_G Model has Chirality at C00Q         (Sohnke SpGr)        S Verify
PLAT850_ALERT_4_G Check Flack Parameter Exact Value 0.00 with s.u.      0.60 Check
PLAT860_ALERT_3_G Number of Least-Squares Restraints .............        72 Note
PLAT912_ALERT_4_G Missing # of FCF Reflections Above STh/L=  0.600         3 Note
PLAT913_ALERT_3_G Missing # of Very Strong Reflections in FCF ....         1 Note
PLAT933_ALERT_2_G Number of HKL-OMIT Records in Embedded .res File        17 Note
PLAT978_ALERT_2_G Number C-C Bonds with Positive Residual Density.         2 Info
```

0 **ALERT level A** = Most likely a serious problem - resolve or explain
0 **ALERT level B** = A potentially serious problem, consider carefully
17 **ALERT level C** = Check. Ensure it is not caused by an omission or oversight
15 **ALERT level G** = General information/check it is not something unexpected

0 ALERT type 1 CIF construction/syntax error, inconsistent or missing data
13 ALERT type 2 Indicator that the structure model may be wrong or deficient
9 ALERT type 3 Indicator that the structure quality may be low
10 ALERT type 4 Improvement, methodology, query or suggestion
0 ALERT type 5 Informative message, check

Validation response form

Please find below a validation response form (VRF) that can be filled in and pasted into your CIF.

```
# start Validation Reply Form
_vrf_STRVA01_2204281_0m
;
PROBLEM: Flack test results are meaningless.
RESPONSE: ...
;
_vrf_PLAT213_2204281_0m
;
PROBLEM: Atom C00V            has ADP max/min Ratio .....        3.2 prolat
RESPONSE: ...
;
_vrf_PLAT220_2204281_0m
;
PROBLEM: NonSolvent   Resd 1  C   Ueq(max)/Ueq(min) Range        3.3 Ratio
RESPONSE: ...
;
_vrf_PLAT222_2204281_0m
;
PROBLEM: NonSolvent Resd 1  H   Uiso(max)/Uiso(min) Range        4.1 Ratio
RESPONSE: ...
;
_vrf_PLAT230_2204281_0m
;
PROBLEM: Hirshfeld Test Diff for    C009     --C00G     .        6.4 s.u.
RESPONSE: ...
;
_vrf_PLAT242_2204281_0m
;
PROBLEM: Low    'MainMol' Ueq as Compared to Neighbors of       O001 Check
RESPONSE: ...
;
_vrf_PLAT250_2204281_0m
;
PROBLEM: Large U3/U1 Ratio for Average U(i,j) Tensor ....        2.1 Note
RESPONSE: ...
;
_vrf_PLAT340_2204281_0m
;
PROBLEM: Low Bond Precision on  C-C Bonds ..............    0.00514 Ang.
RESPONSE: ...
;
_vrf_PLAT905_2204281_0m
;
PROBLEM: Negative K value in the Analysis of Variance ...    -11.836 Report
RESPONSE: ...
;
_vrf_PLAT910_2204281_0m
;
PROBLEM: Missing # of FCF Reflection(s) Below Theta(Min).          5 Note
RESPONSE: ...
;
_vrf_PLAT911_2204281_0m
;
```

```
PROBLEM: Missing FCF Refl Between Thmin & STh/L=    0.600         10 Report
RESPONSE: ...
;
# end Validation Reply Form
```

It is advisable to attempt to resolve as many as possible of the alerts in all categories. Often the minor alerts point to easily fixed oversights, errors and omissions in your CIF or refinement strategy, so attention to these fine details can be worthwhile. In order to resolve some of the more serious problems it may be necessary to carry out additional measurements or structure refinements. However, the purpose of your study may justify the reported deviations and the more serious of these should normally be commented upon in the discussion or experimental section of a paper or in the "special_details" fields of the CIF. checkCIF was carefully designed to identify outliers and unusual parameters, but every test has its limitations and alerts that are not important in a particular case may appear. Conversely, the absence of alerts does not guarantee there are no aspects of the results needing attention. It is up to the individual to critically assess their own results and, if necessary, seek expert advice.

Publication of your CIF in IUCr journals

A basic structural check has been run on your CIF. These basic checks will be run on all CIFs submitted for publication in IUCr journals (*Acta Crystallographica*, *Journal of Applied Crystallography*, *Journal of Synchrotron Radiation*); however, if you intend to submit to *Acta Crystallographica Section C* or *E* or *IUCrData*, you should make sure that full publication checks are run on the final version of your CIF prior to submission.

Publication of your CIF in other journals

Please refer to the *Notes for Authors* of the relevant journal for any special instructions relating to CIF submission.

PLATON version of 28/11/2022; check.def file version of 28/11/2022

Datablock 2204281_0m - ellipsoid plot

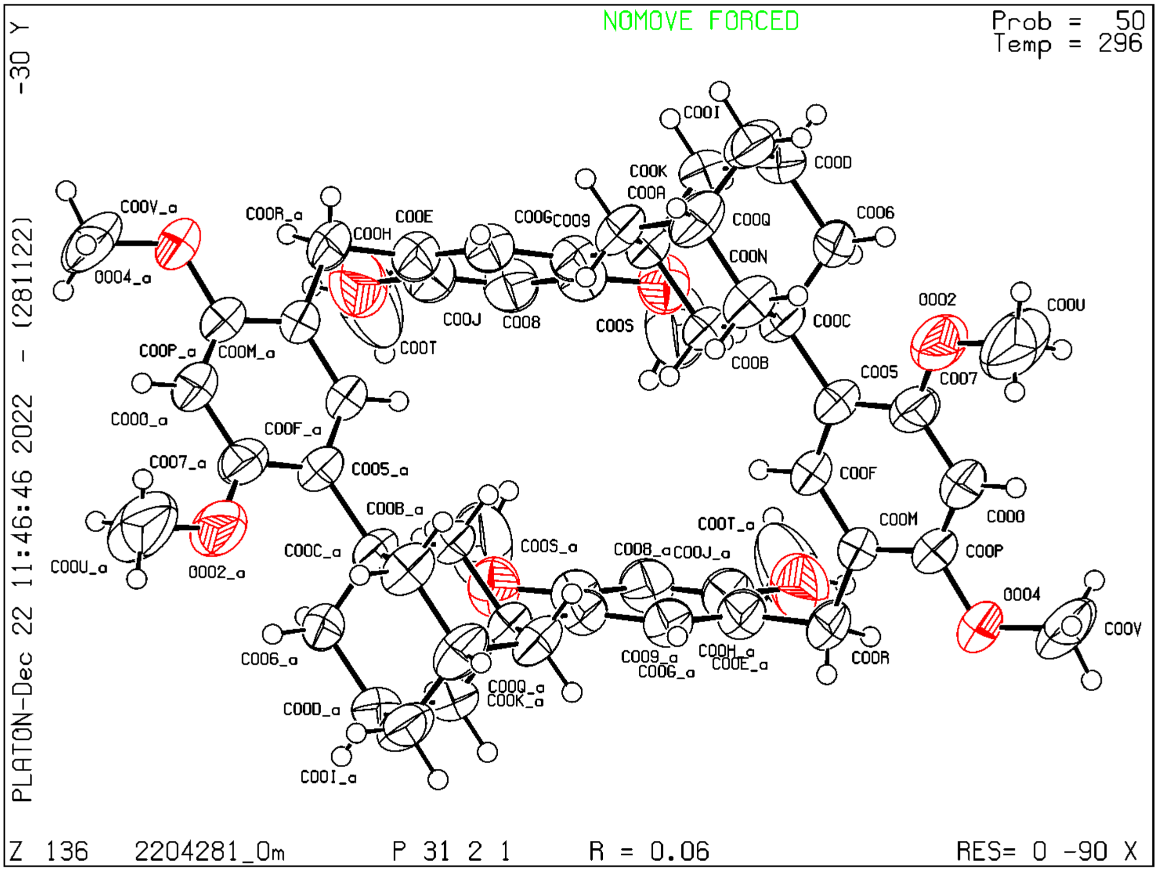

Supplement: Supplementary file 1 [file molecules-29-00719-s001.zip › 2204281_0m checkcif.pdf]
