# Supplementary material for: Keto-Adamantane-Based Macrocycle Crystalline Supramolecular Assemblies Showing Selective Vapochromism to Tetrahydrofuran
Source: Molecules. 2024 Feb 4;29(3):719. doi: 10.3390/molecules29030719 (PMC10856198; doi:10.3390/molecules29030719)
Supplement: Supplementary file 1 [file molecules-29-00719-s001.zip › Supporting information.pdf]

Supporting Information

Keto-adamantane-based macrocycle crystalline supramolecular assemblies showing selective vapochromism to THF

Zun-Hua Li[1], Ying-Zi Tan[1], Man-Hua Ding[1]*, Lin-Li Tang[1] and Fei Zeng[1,]*

Contents

1. **Materials and Methods...3**
2. **Synthesis of New compounds...3**
3. **^{1}H NMR and ^{13}C NMR Spectral of New compounds. 5**
4. **UV-vis spectral of 3 and DDQ 9**
5. **Characterization of 3@DDQ 10**
6. **Vapor adsorption experiments 11**
7. **Complexation between 3 and DDQ in THF...21**
8. **Crystal data 21**

1. Materials and Methods.

All reactions were carried out with oven-dried glassware. Commercial reagents were used without further purification. Flash column chromatography was performed on 100-200 mesh silica gel. ^{1}H NMR, ^{13}C NMR spectra were recorded on a Bruker DMX400 NMR spectrometer. Melting points were determined using WRR melting point apparatus and were uncorrected. High Resolution atmospheric-pressure chemical ionization mass spectra (APCI-MS) were determined by Bruker Daltonics. Inc, APEX II.

Powder X-ray diffraction (PXRD) data were collected on a Rigaku Ultimate-IV X-ray diffractometer operating at 40 kV/30 mA using the Cu Kα line (λ = 1.5418 Å). Data were measured over the range of 5-45° in 5°/min steps over 8 min.

Thermogravimetric analysis (TGA) was carried out using a Q5000IR analyzer (TA Instruments) with an automated vertical overhead thermobalance. The samples were heated at 10 °C/min using N2 as the protective gas.

Vapochromic experiments. An open 2 mL vial containing 10 mg of **3**@DDQ was placed in a sealed 20 mL vial containing 1 mL of each vapor solution. **3**@DDQ powders were exposed under saturated vapor pressure in the closed vessel at room temperature. Obvious color changes were observed after 12 hours.

Sample preparation of 3@DDQ. 3 (1.085 g) and DDQ (0.282 g) were dissolved in 100 mL CH_2Cl_2, and then rapid evaporation of the solution under vacuum at 50 °C afforded dark green co-crystals **3**@DDQ.

2. Synthesis of New compounds.

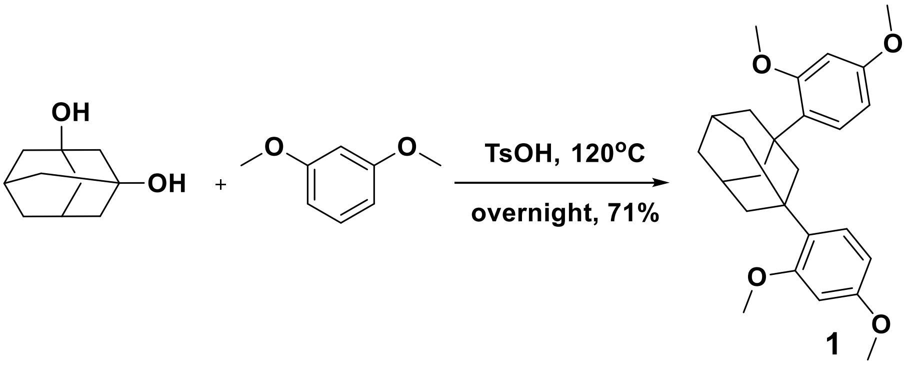

1

Compound 1 A mixture of 1,3-dihydroxyadamantane (1680 mg, 10 mmol), 1,3-dimethoxy-benzene (9660 mg, 70 mmol) and TsOH (860 mg, 5 mmol) in a flask was stirred at 120 °C for overnight under N_2. After quenching the reaction with water (100 ml), resulting mixture was extracted with

dichloromethane (3 × 50 mL) and then washed with water and brine successively. The organic layer was dried over anhydrous Na_2SO_4 and evaporated. The residue was purified by column chromatography with dichloromethane/ Petroleum ether as eluent to afford compound **1** (2900 mg, yield 71%) as white solid. ^{1}H NMR (400 MHz, $CDCl_3$) δ 7.19 (d, J = 8.4 Hz, 2H), 6.53-6.43 (m, 4H), 3.82 (s, 12H), 2.40 (d, J = 10.6 Hz, 2H), 2.25-2.02 (m, 10H), 1.79 (s, 2H). ^{13}C NMR (101 MHz, $CDCl_3$) δ 159.7, 158.8, 131.2, 126.9, 103.4, 99.7, 55.3, 55.0, 43.6, 40.6, 40.4, 37.7, 37.4, 36.7, 29.9, 29.9. HRMS (APCI) m/z: $[M+H]^+$ calcd for $C_{26}H_{33}O_4$, 409.2379; found, 409.2385. Anal calcd for $C_{26}H_{32}O_4$: C, 76.44; H, 7.90. Found: C, 76.41; H, 7.92.

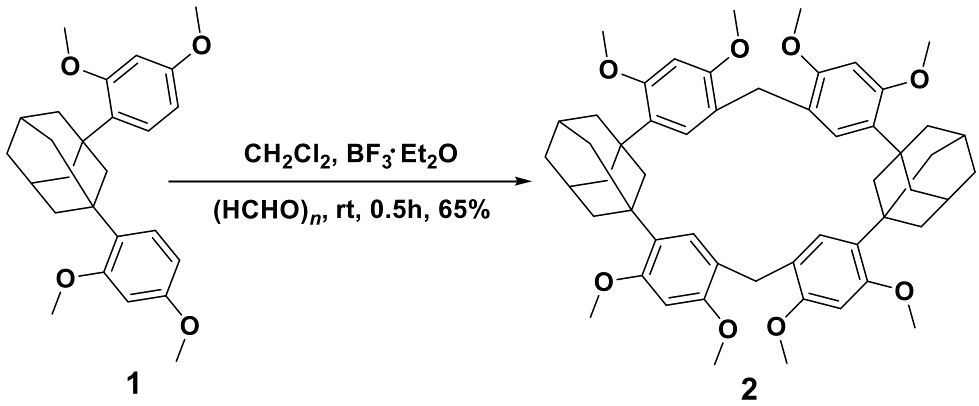

Compound 2 To a mixture of **3** (820 mg, 2.0 mmol) and paraformaldehyde (180 mg, 6.0 mmol) in dichloromethane (150 mL) was added catalytic amount of boron trifluoride diethyl etherate (0.3 mL, 2.4 mmol). The mixture was stirred at room temperature for 0.5 h. Then the reaction was quenched by the addition of 150 mL water. The organic layer was separated and dried with anhydrous $MgSO_4$. The solvent was removed in vacuo and the residue was separated by column chromatography on silica gel (eluent: 3:1 DCM/Petroleum ether) to give **2** (546 mg, 65%) as yellow solids. ^{1}H NMR (400 MHz, $CDCl_3$) δ 6.49 (s, 4H), 6.42 (s, 4H), 3.75 (d, J = 3.4 Hz, 28H), 2.13-1.96 (m, 12H), 1.80-1.57 (m, 16H). ^{13}C NMR (101 MHz, $CDCl_3$) δ 157.8, 156.2, 130.5, 127.9, 120.1, 96.4, 55.6, 55.5, 44.8, 40.1, 37.1, 36.2, 29.7, 28.5. HRMS (APCI) m/z: $[M+H]^+$ calcd for $C_{54}H_{65}O_8$, 841.4679; found, 841.4675. Anal calcd for $C_{54}H_{64}O_8$: C, 77.11; H, 7.67. Found: C, 77.09; H, 7.68.

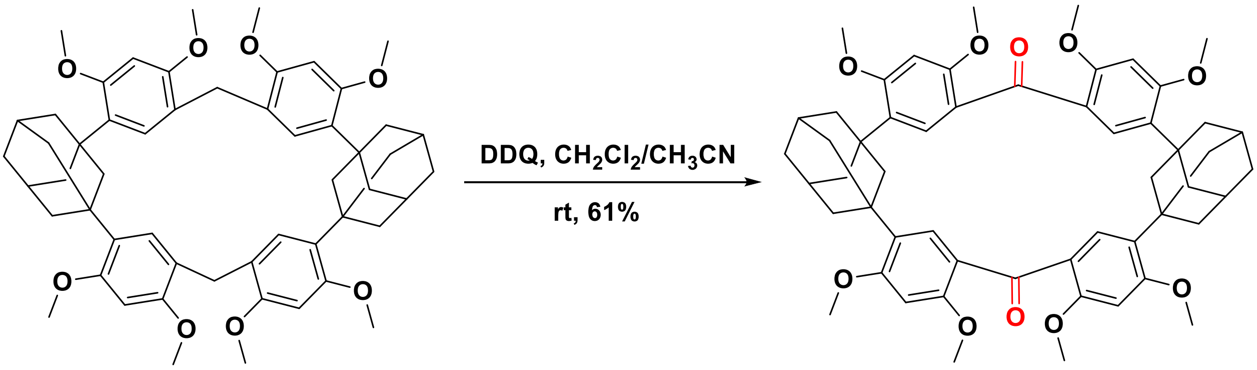

Compound 3 A mixture of **2** (4200 mg, 5 mmol) and DDQ (11300 mg, 50 mmol) in CH_2Cl_2/CH_3CN (V/V=1:1) was stirred at room temperature for overnight. The mixture was evaporated and purified by column chromatography with dichloromethane/CH_3OH as eluent to afford compound **3** (2650 mg, yield 61%) as white solid. ^1H NMR (400 MHz, $CDCl_3$) δ 7.06 (s, 4H), 6.50 (s, 4H), 3.90 (d, J = 16.5 Hz, 24H), 2.10 (s, 6H), 1.70 (s, 6H), 1.59-1.53 (m, 16H). ^{13}C NMR (101 MHz, $CDCl_3$) δ193.8, 155.0, 129.1, 97.0, 56.5, 55.2, 45.2, 36.7, 36.0, 29.4. HRMS (APCI) m/z: $[M+H]^+$ calcd for $C_{54}H_{61}O_{10}$, 869.4265; found, 869.4271. Anal calcd for $C_{54}H_{60}O_{10}$: C, 74.63; H, 6.96. Found: C, 74.64; H, 6.98.

3. ^1H NMR and ^{13}C NMR Spectral of New compounds.

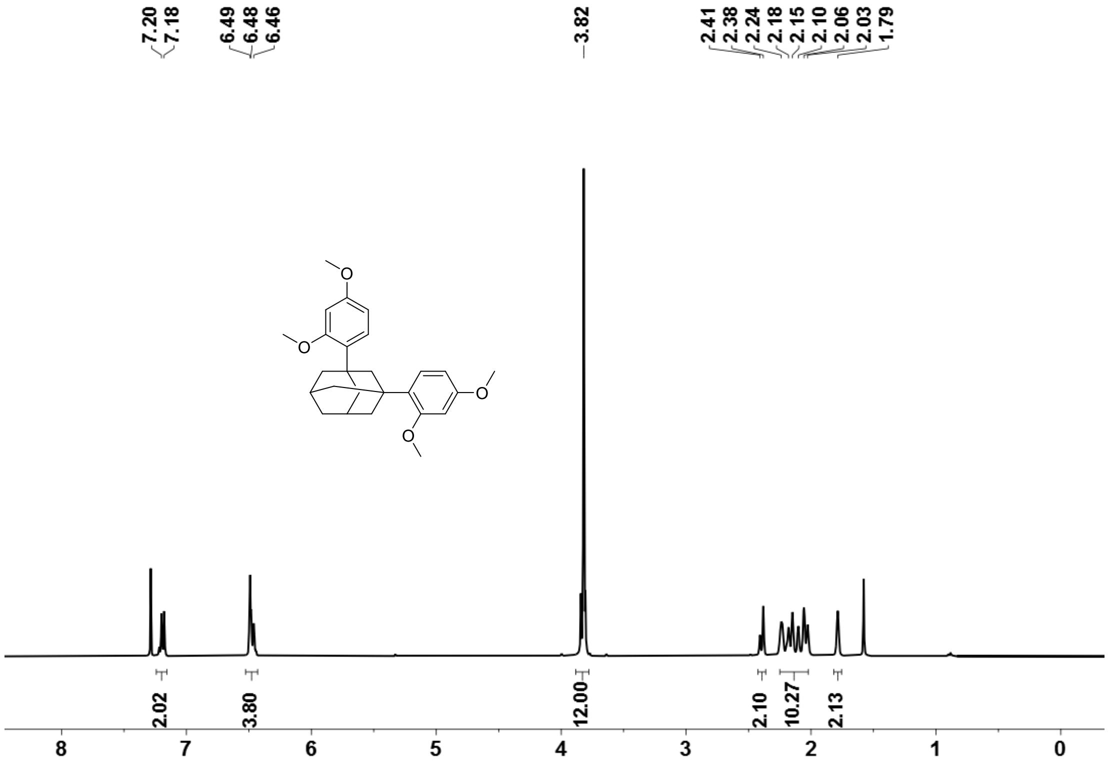

Figure S1. ^1H NMR spectrum (400 MHz, $CDCl_3$, 298K) of **1**

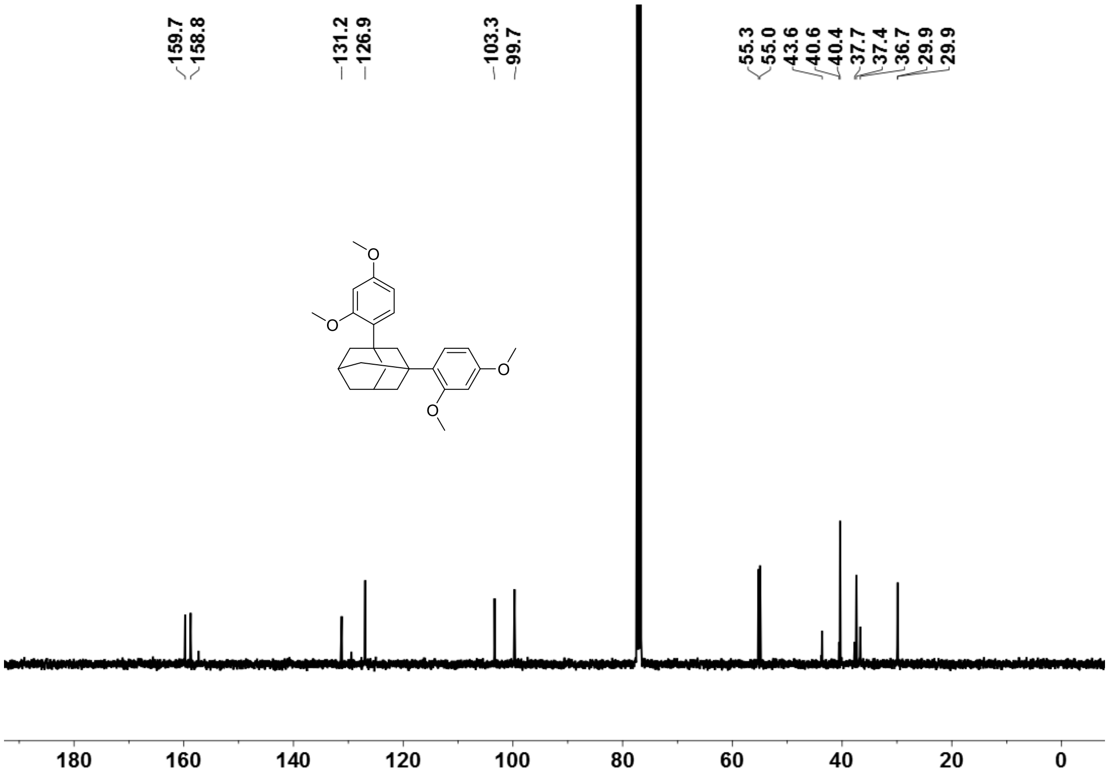

Figure S2.^{13}C NMR spectrum (101 MHz, $CDCl_3$, 298K) of **1**

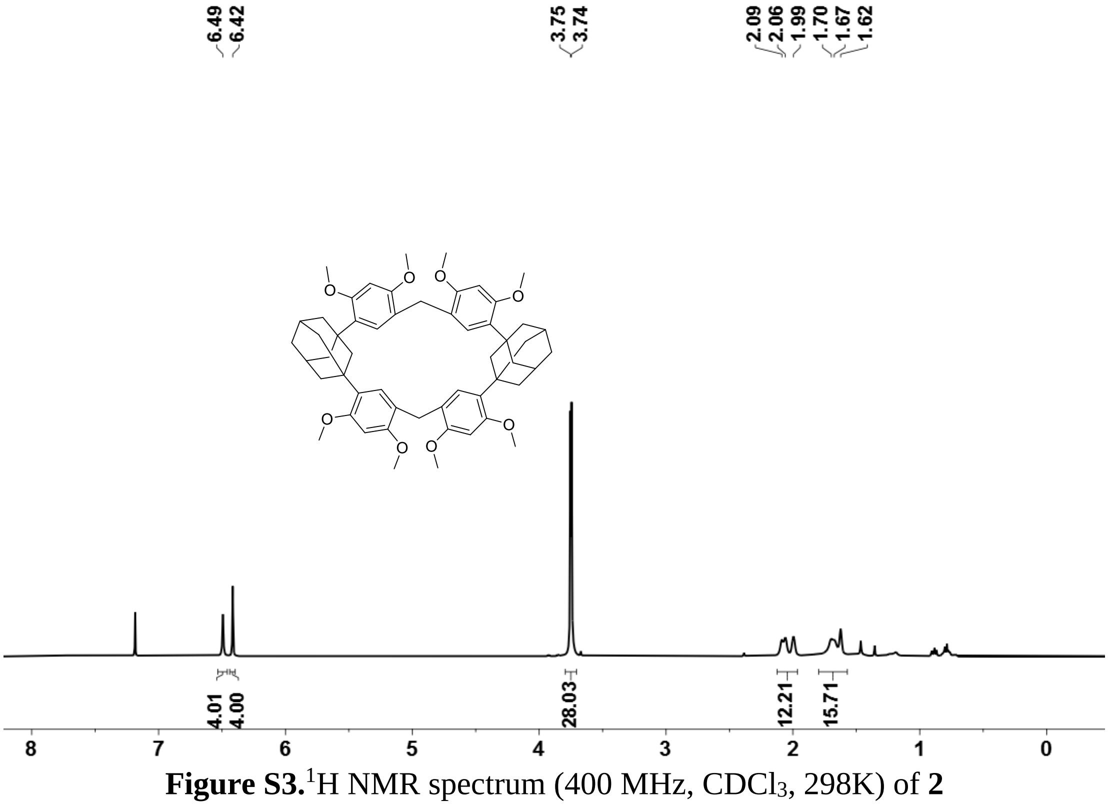

Figure S3.^{1}H NMR spectrum (400 MHz, $CDCl_3$, 298K) of **2**

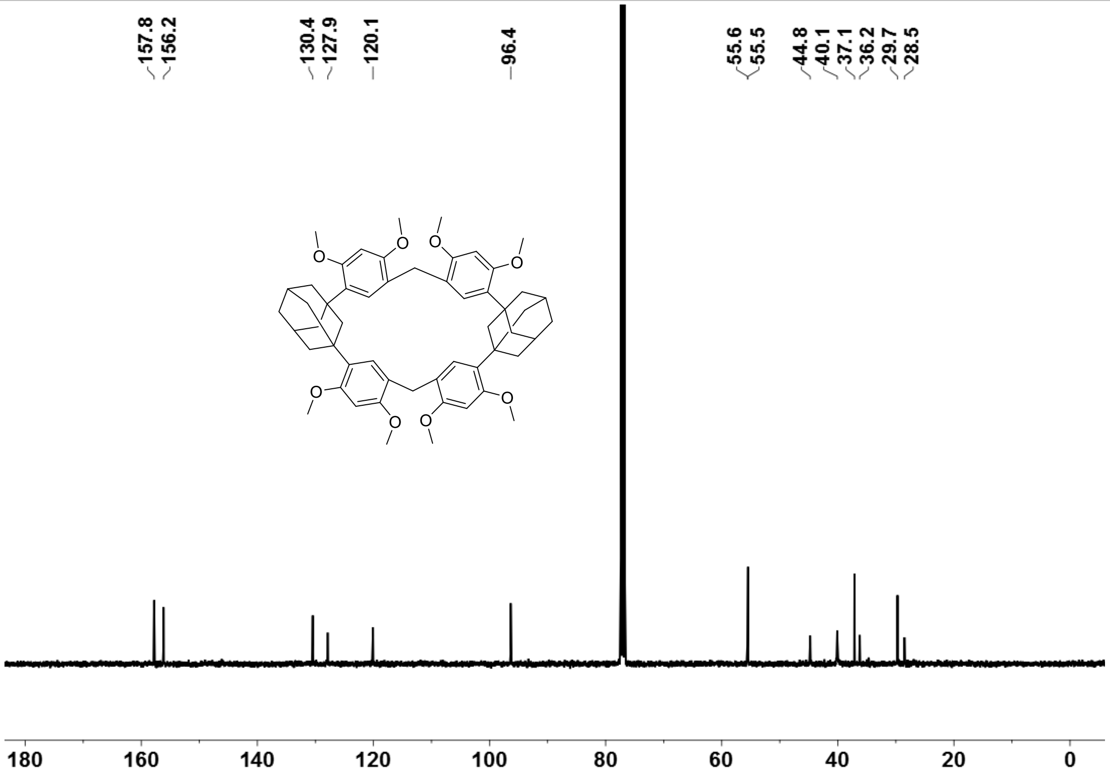

Figure S4. ^{13}C NMR spectrum (101 MHz, $CDCl_3$, 298K) of **2**

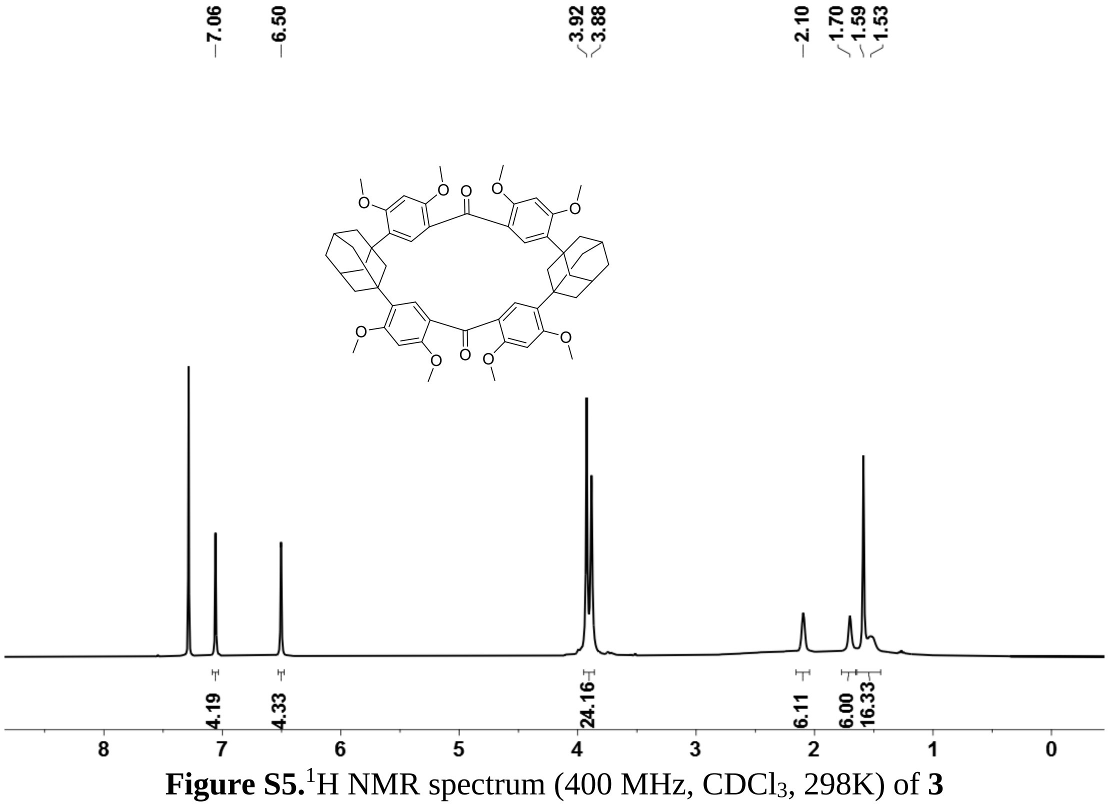

Figure S5. ^{1}H NMR spectrum (400 MHz, $CDCl_3$, 298K) of **3**

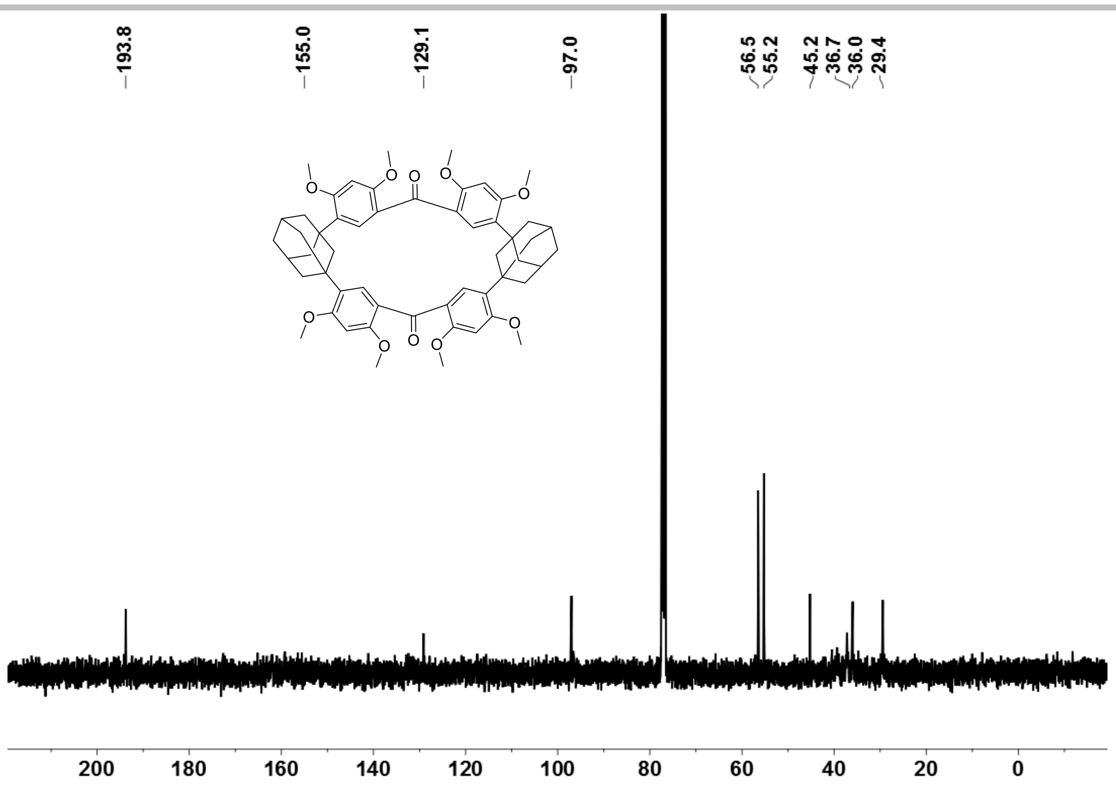

Figure S6. ^{13}C NMR spectrum (101 MHz, $CDCl_3$, 298K) of **3**

4. UV-vis spectral of 3 and DDQ

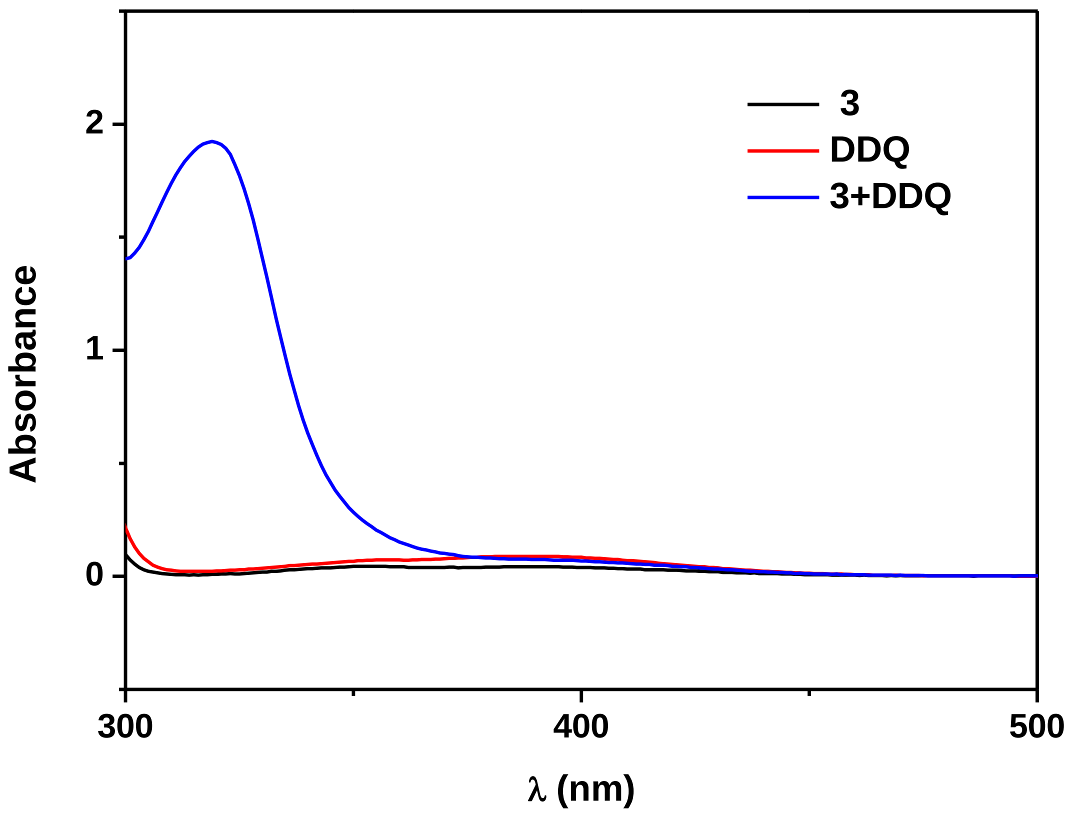

Figure S7. Absorption spectra of 0.1 mM **3**, 0.1 mM DDQ and an equimolar mixture of **3** (0.1 Mm) and DDQ (0.1 mM) in $CHCl_3$.

5. Characterization of 3@DDQ

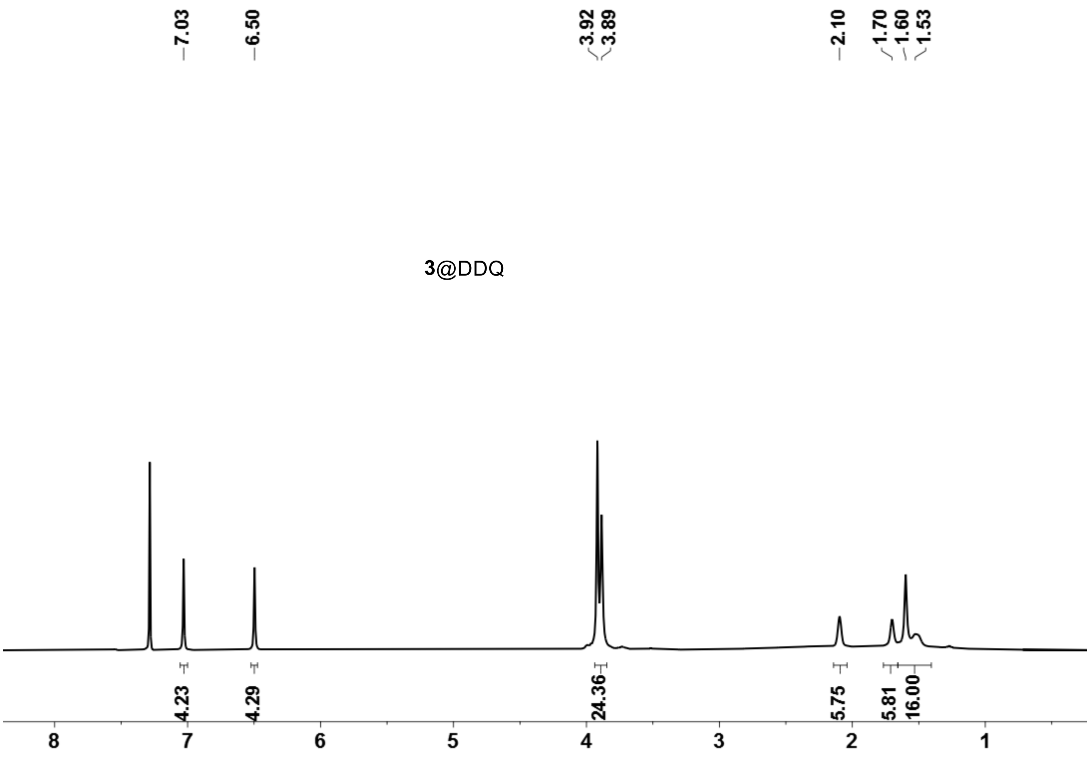

Figure S8. ^{1}H NMR spectrum (400 MHz, $CDCl_3$, 298K) of **1**@DDQ that prepared by rapid evaporation of the CH_2Cl_2 solution of **3** and DDQ at 1:1 molar ratio.

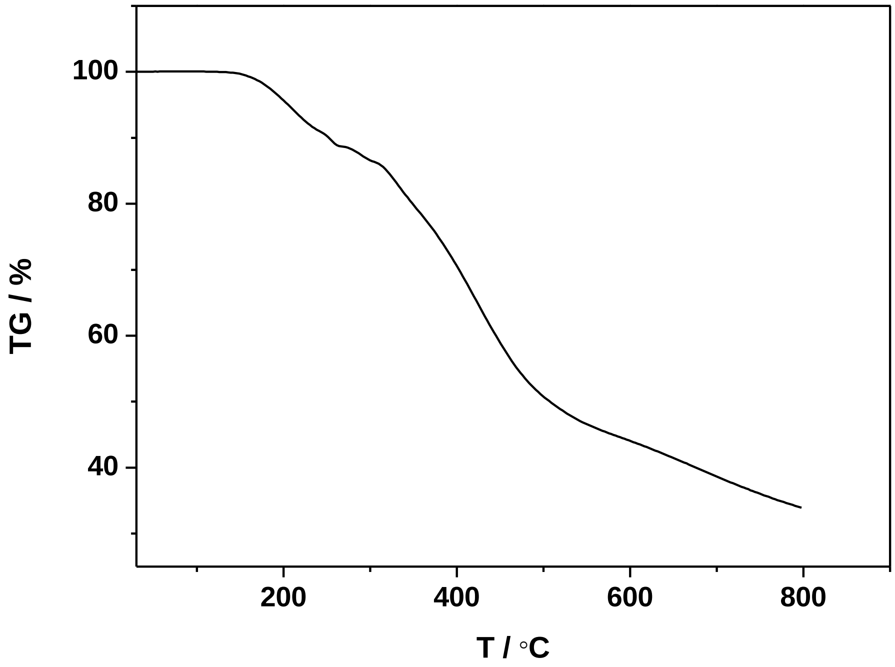

Figure S9. Thermogravimetric analysis of **3@**DDQ that prepared by rapid evaporation of the CH_2Cl_2 solution of **3** and DDQ at 1:1 molar ratio.

6. Vapor adsorption experiments

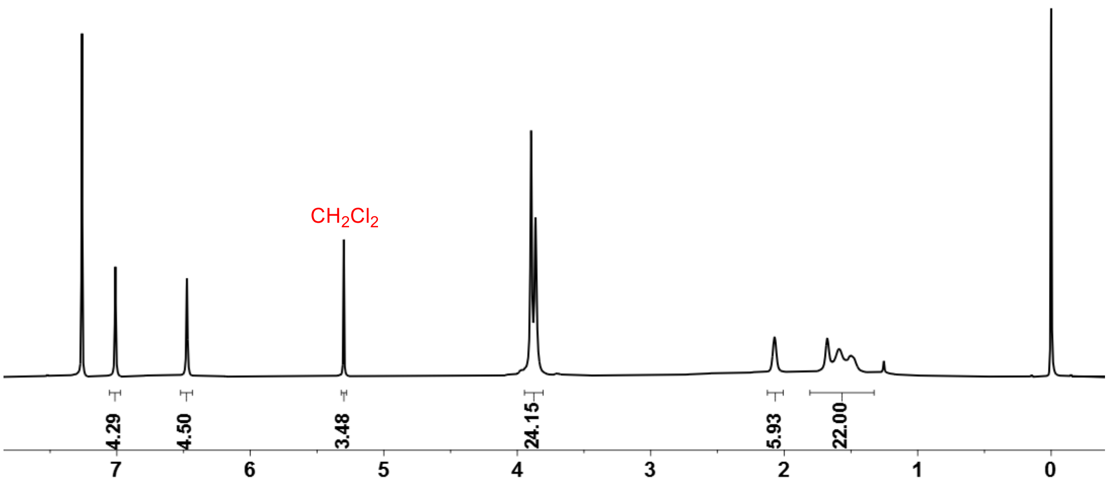

Figure S10. ^{1}H NMR spectrum (400 MHz, $CDCl_3$, 293 K) of **3**@DDQ after sorption of CH_2Cl_2 vapor.

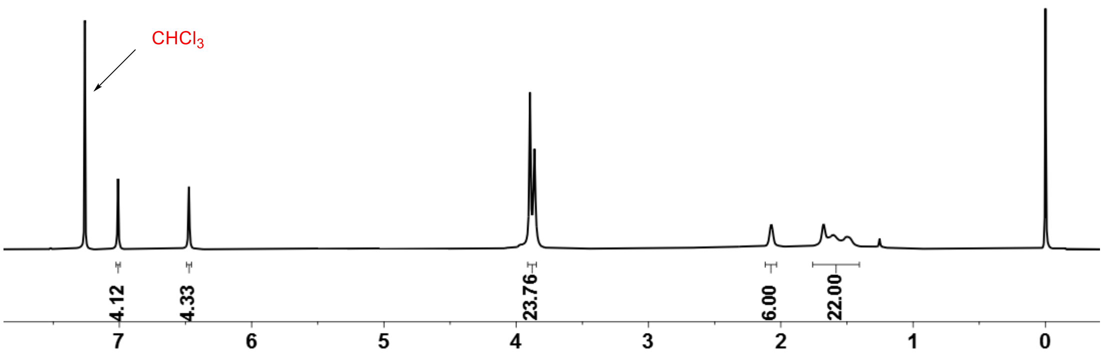

Figure S11. ^{1}H NMR spectrum (400 MHz, $CDCl_3$, 293 K) of **3**@DDQ after sorption of $CHCl_3$ vapor.

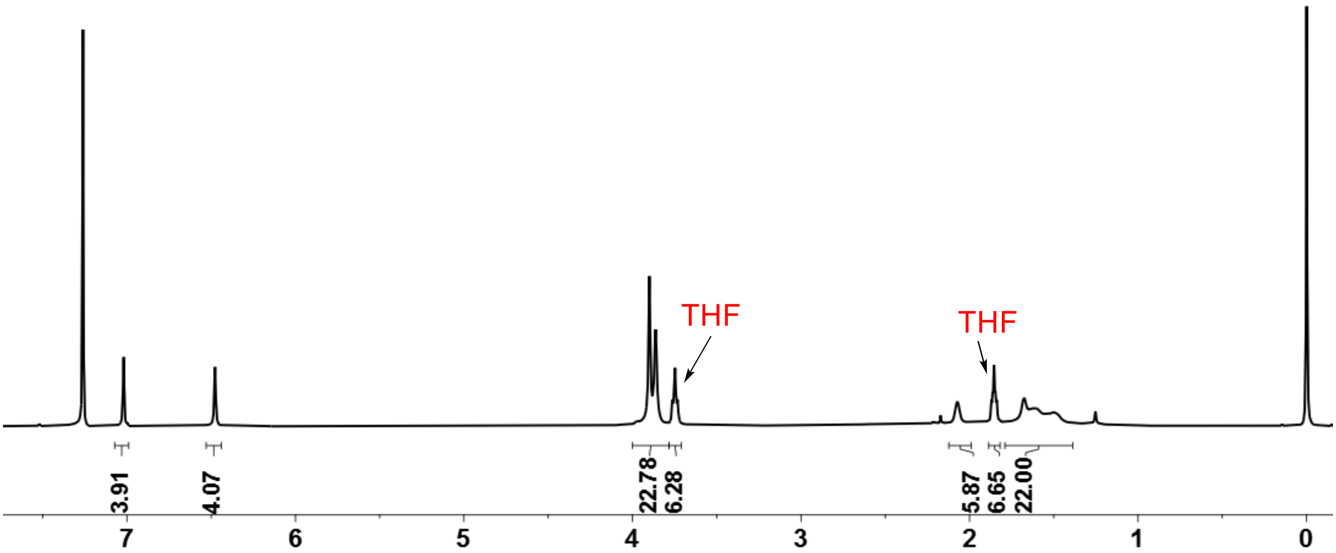

Figure S12. ^{1}H NMR spectrum (400 MHz, $CDCl_3$, 293 K) of **3**@DDQ after sorption of THF vapor.

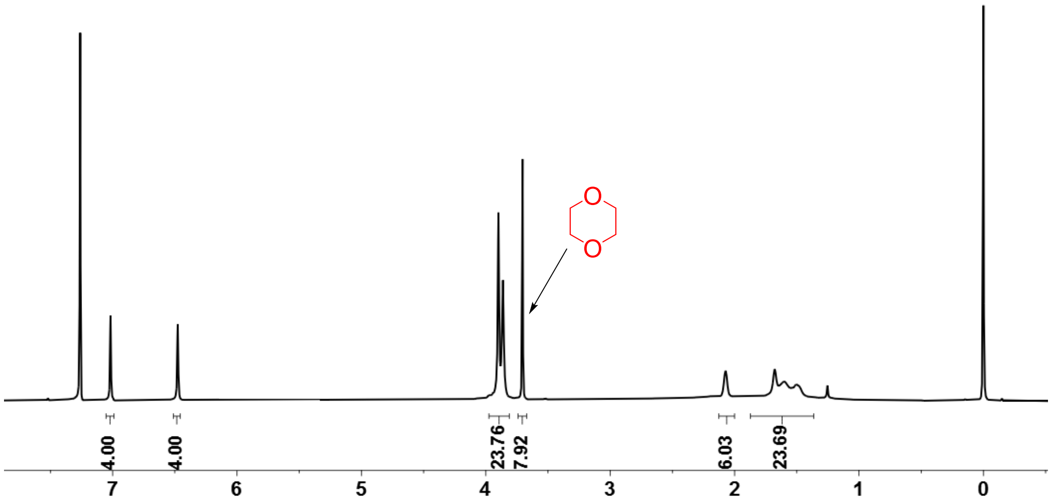

Figure S13. ^{1}H NMR spectrum (400 MHz, $CDCl_3$, 293 K) of **3**@DDQ after sorption of 1,4-dioxane vapor.

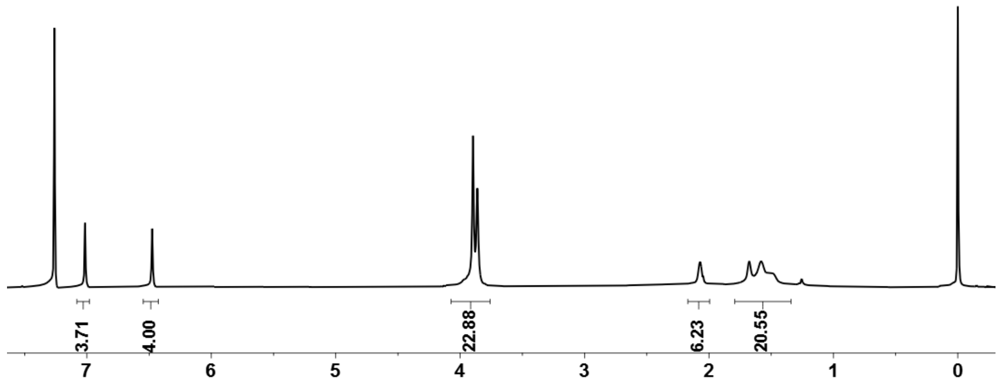

Figure S14. ^{1}H NMR spectrum (400 MHz, $CDCl_3$, 293 K) of **3**@DDQ after sorption of EtOAc vapor.

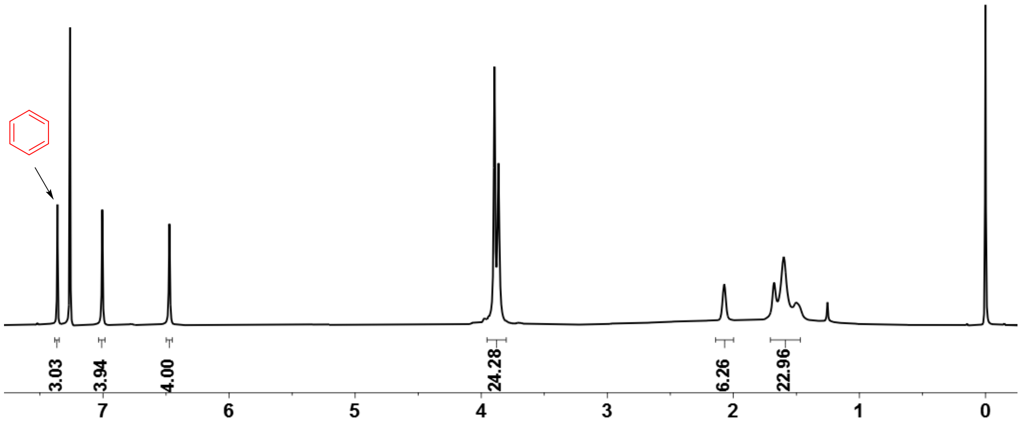

Figure S15. ^{1}H NMR spectrum (400 MHz, $CDCl_3$, 293 K) of **3**@DDQ after sorption of Benzene vapor.

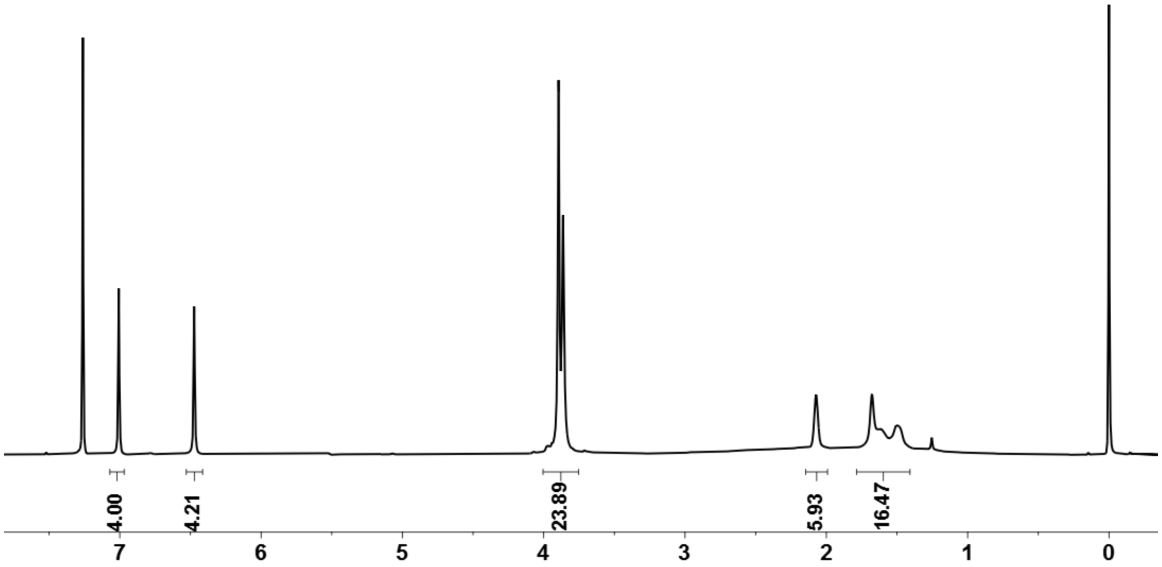

Figure S16. ^{1}H NMR spectrum (400 MHz, $CDCl_3$, 293 K) of **3**@DDQ after sorption of *n*-Hexane vapor.

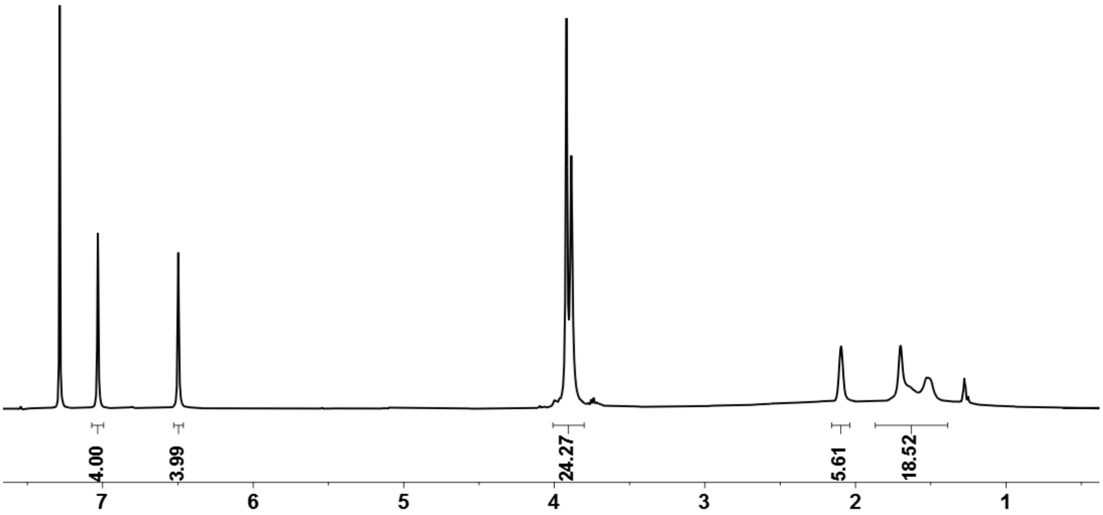

Figure S17. ^{1}H NMR spectrum (400 MHz, $CDCl_3$, 293 K) of **3**@DDQ after sorption of EtOH vapor.

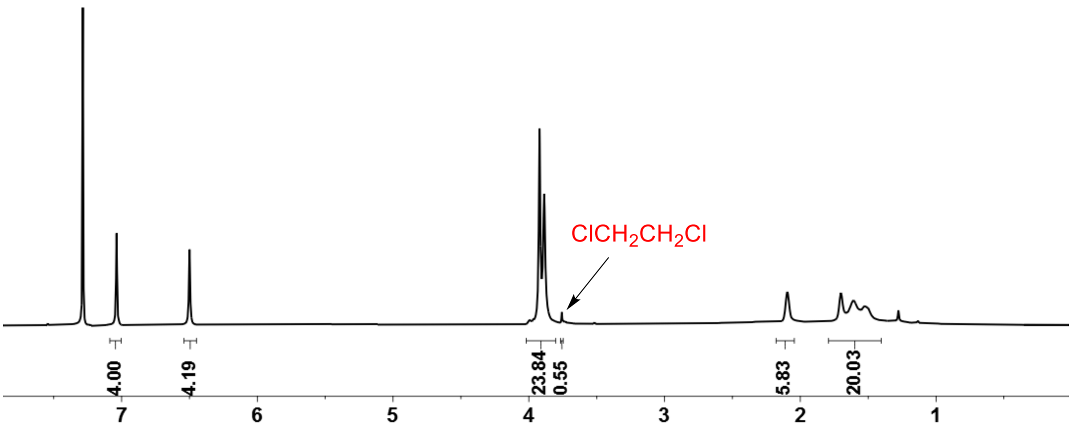

Figure S18. ^{1}H NMR spectrum (400 MHz, $CDCl_3$, 293 K) of **3**@DDQ after sorption of $ClCH_2CH_2Cl$ vapor.

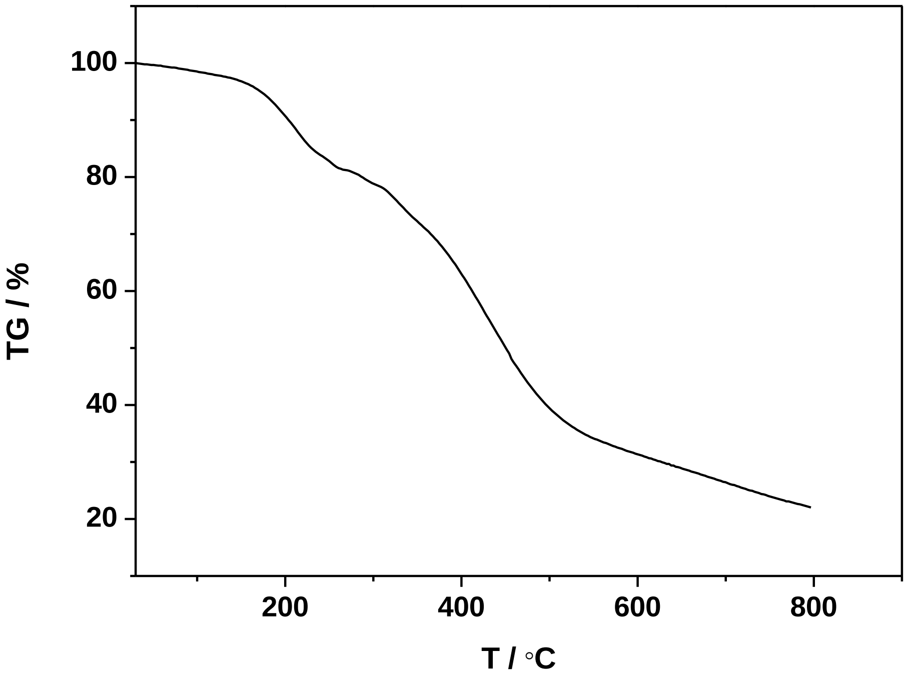

Figure S19. Thermogravimetric analysis of **3@**DDQ after sorption of CH_2Cl_2 vapor.

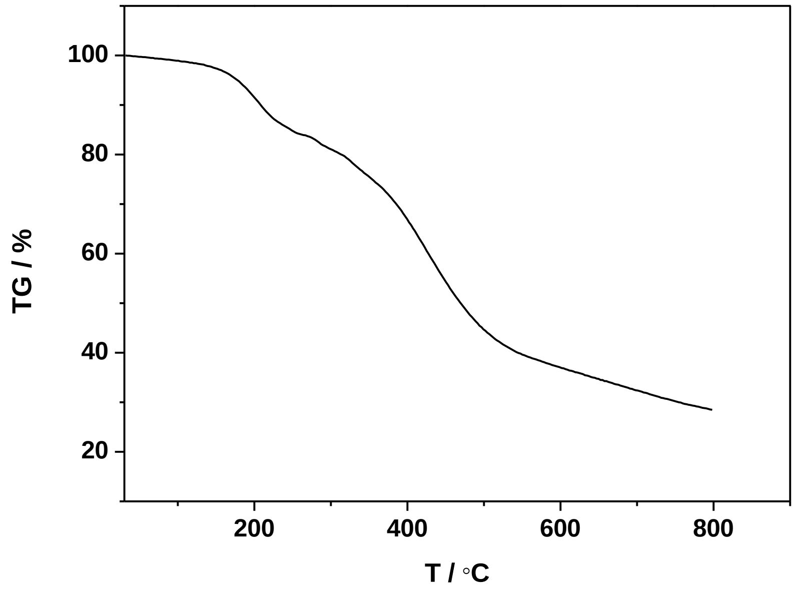

Figure S20. Thermogravimetric analysis of **3@**DDQ after sorption of $CHCl_3$ vapor.

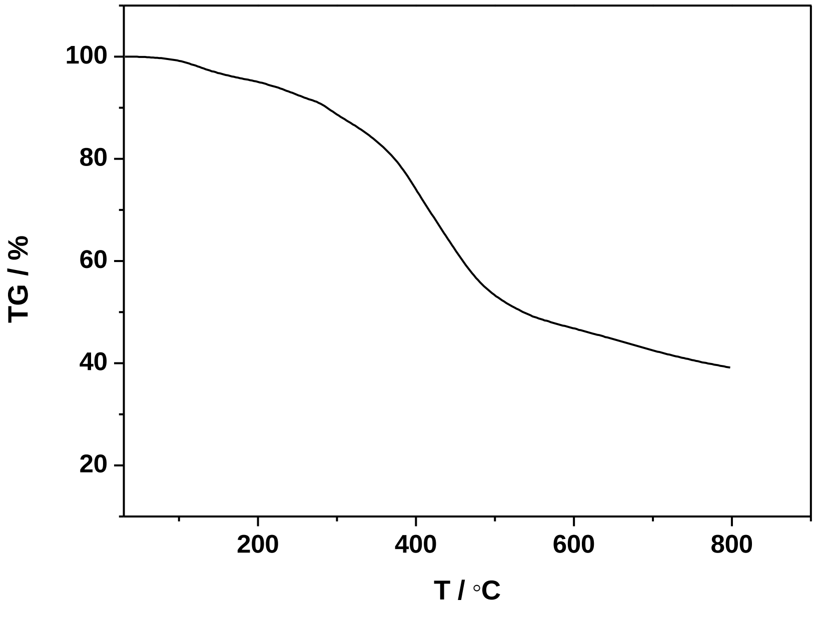

Figure S21. Thermogravimetric analysis of **3@**DDQ after sorption of THF vapor.

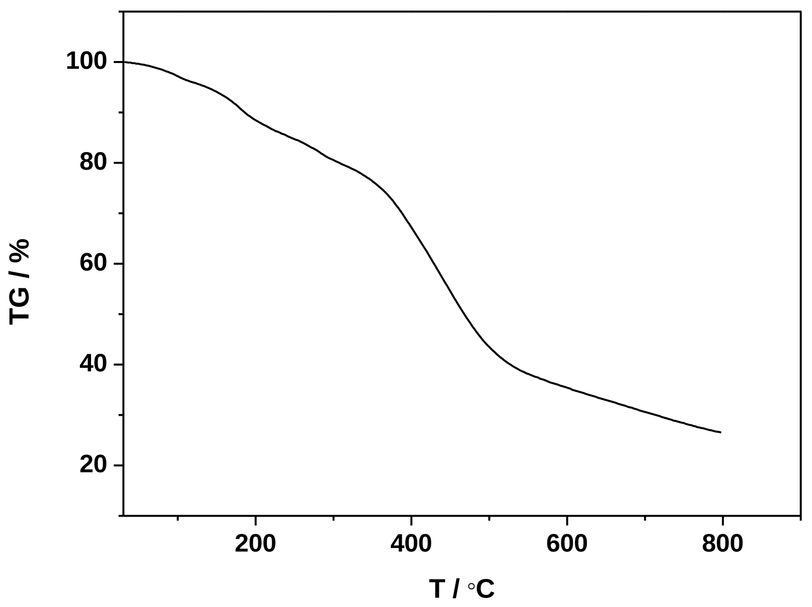

Figure S22. Thermogravimetric analysis of **3**@DDQ after sorption of 1,4-dioxane vapor.

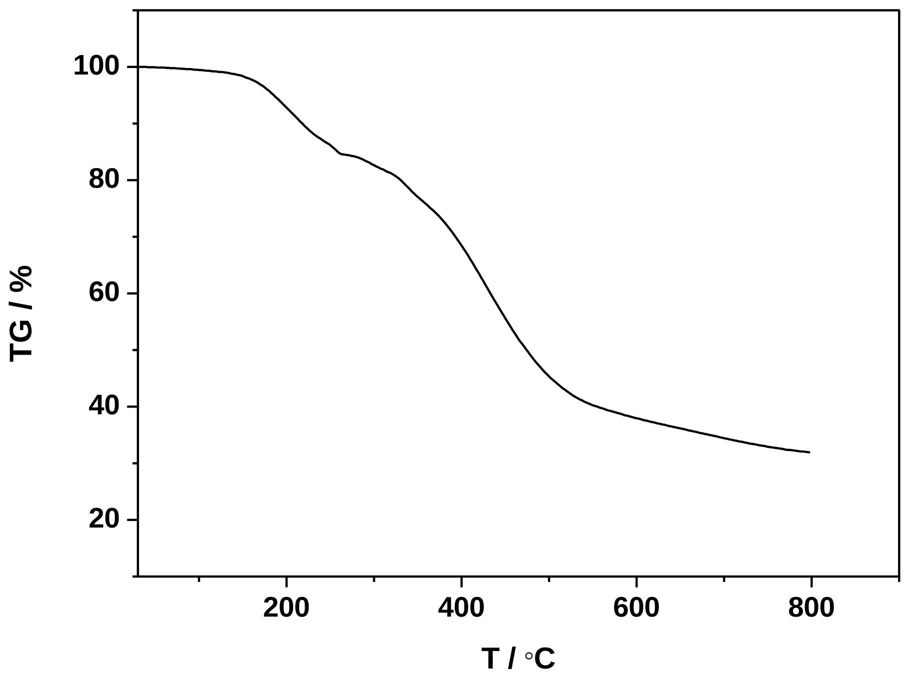

Figure S23. Thermogravimetric analysis of **3**@DDQ after sorption of EtOAc vapor.

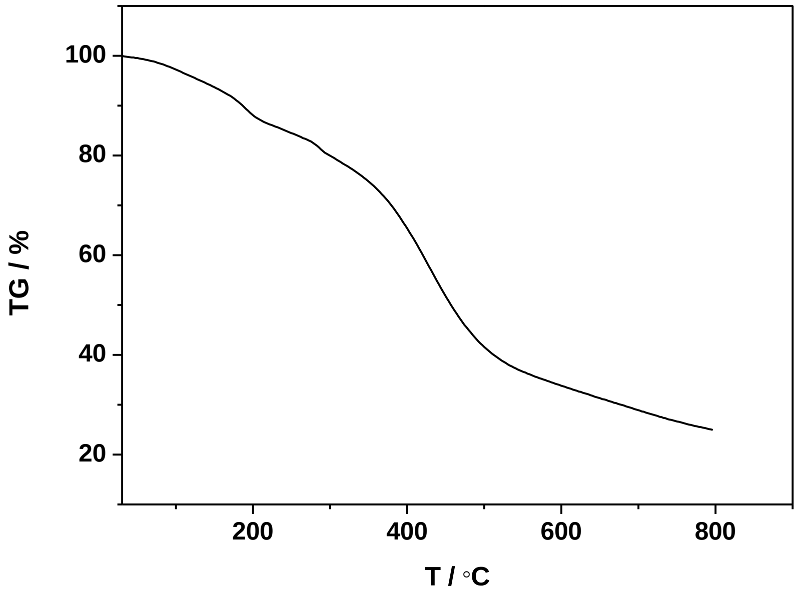

Figure S24. Thermogravimetric analysis of **3@**DDQ after sorption of Benzene vapor.

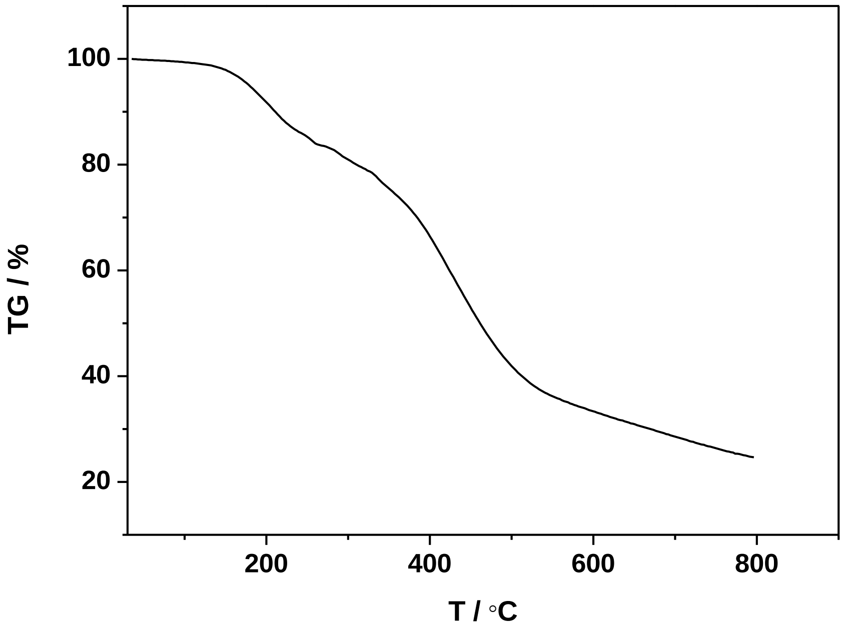

Figure S25. Thermogravimetric analysis of **3@**DDQ after sorption of *n*-Hexane vapor.

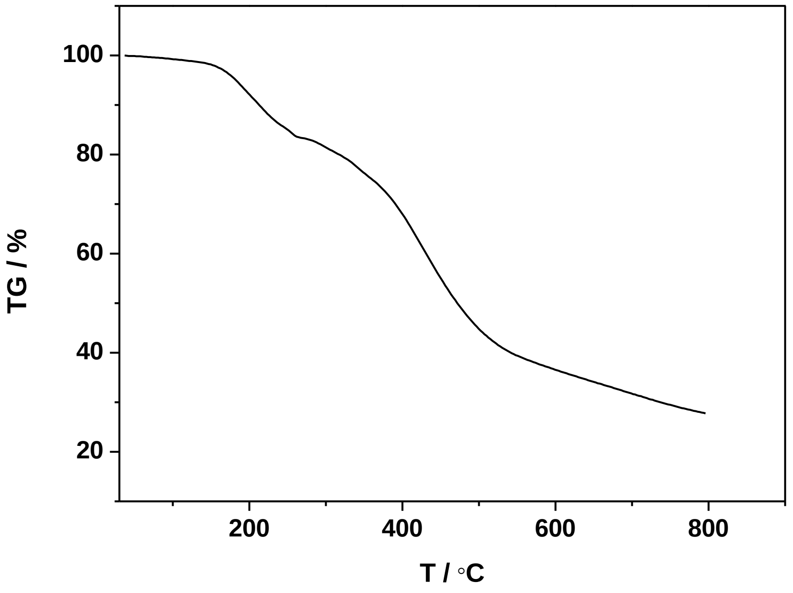

Figure S26. Thermogravimetric analysis of **3@**DDQ after sorption of EtOH vapor.

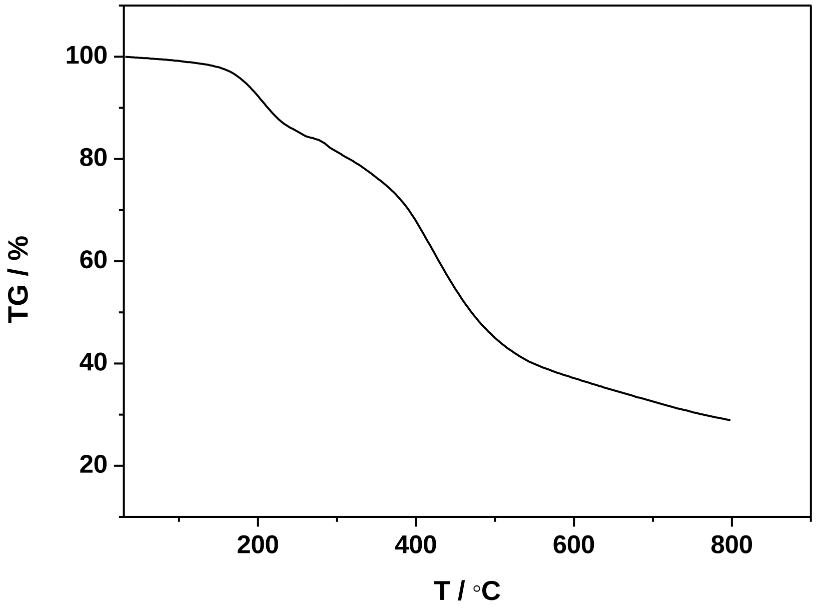

Figure S27. Thermogravimetric analysis of **3@**DDQ after sorption of $ClCH_2CH_2Cl$ vapor.

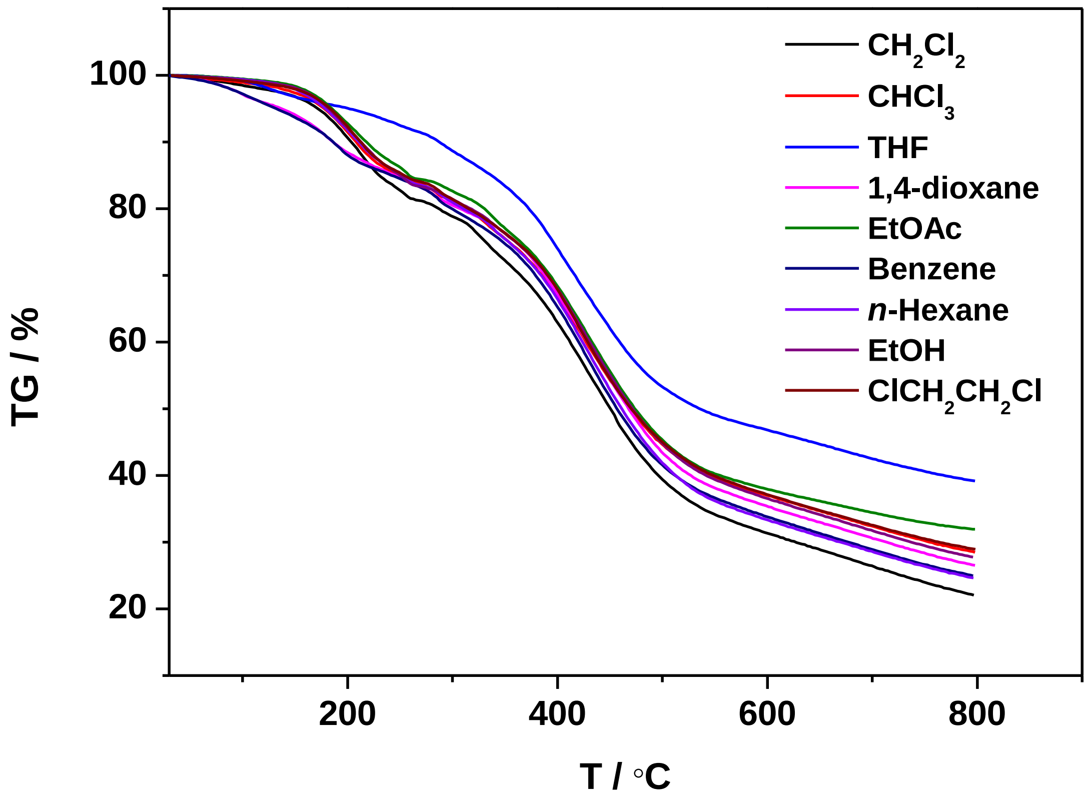

Figure S27-1. Thermogravimetric analysis of **3**@DDQ after sorption of vapors.

7. Complexation between 3 and DDQ in THF

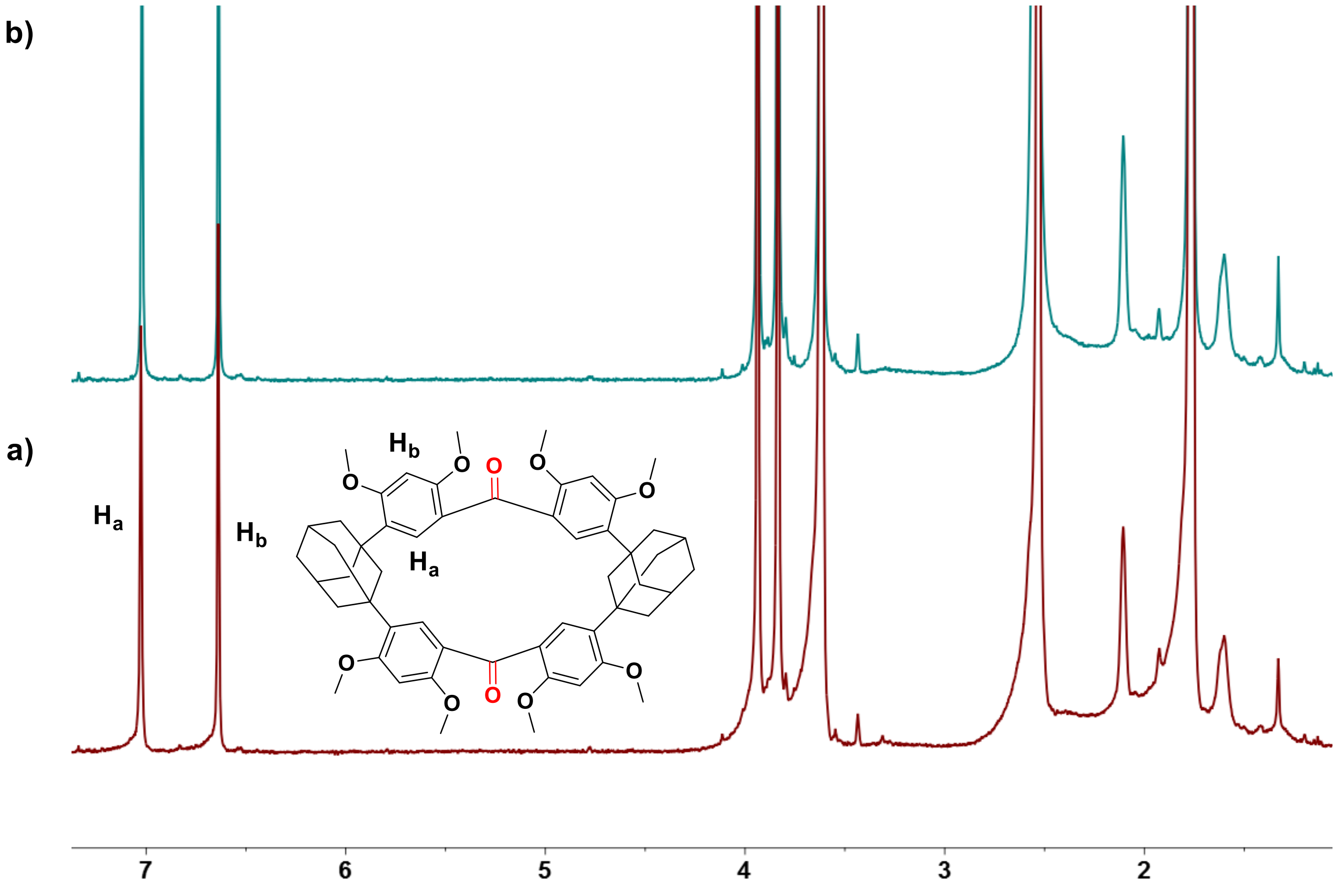

Figure S28. Partial ^{1}H NMR spectra (400 MHz, THF-*d8*, 298 K) of (a) free **3**, (b) **3** and 1.0 equiv. of DDQ. [**3**]$_0$ = 4.0 mM.

8. Crystal data

Crystal data and structure refinement for 2.

Empirical formula	$C_{54}H_{64}O_8$
Formula weight	841.05
Temperature/K	296.15
Crystal system	trigonal
Space group	$P3_121$
a/Å	21.174(3)
b/Å	21.174(3)
c/Å	13.5099(19)
α/°	90

β/°	90
γ/°	120
Volume/Å^3	5245.7(17)
Z	3
ρ_{calc}g/cm^3	0.799
μ/mm^{-1}	0.053
F(000)	1356.0
Crystal size/mm^3	0.12 × 0.1 × 0.09
Radiation	MoKα (λ = 0.71073)
2Θ range for data collection/°	4.888 to 54.962
Index ranges	$-27 \le h \le 27$, $-27 \le k \le 26$, $-17 \le l \le 17$
Reflections collected	53176
Independent reflections	7981 [R_{int} = 0.0676, R_{sigma} = 0.0550]
Data/restraints/parameters	7981/72/284
Goodness-of-fit on F^2	0.974
Final R indexes [I>=2σ (I)]	R_1 = 0.0590, wR_2 = 0.1418
Final R indexes [all data]	R_1 = 0.1165, wR_2 = 0.1670
Largest diff. peak/hole / e Å^{-3}	0.20/-0.15
Flack parameter	0.0(6)

Crystal data and structure refinement for 3.

Empirical formula	$C_{56}H_{64}Cl_4O_{10}$
Formula weight	1038.87
Temperature/K	296.15
Crystal system	triclinic
Space group	P-1
a/Å	14.54(5)
b/Å	15.25(5)
c/Å	15.42(5)
α/°	114.04(4)
β/°	99.38(4)
γ/°	112.59(4)
Volume/Å^3	2667(15)
Z	2
ρ_{calc}g/cm^3	1.294
μ/mm^{-1}	0.279
F(000)	1096.0
Crystal size/mm^3	0.2 × 0.18 × 0.16
Radiation	MoKα (λ = 0.71073)
2Θ range for data collection/°	3.126 to 50
Index ranges	-17 ≤ h ≤ 17, -18 ≤ k ≤ 18, -18 ≤ l ≤ 18
Reflections collected	25065
Independent reflections	9378 [R_{int} = 0.0960, R_{sigma} = 0.1313]
Data/restraints/parameters	9378/256/639
Goodness-of-fit on F^2	0.951
Final R indexes [I>=2σ (I)]	R_1 = 0.0908, wR_2 = 0.2400
Final R indexes [all data]	R_1 = 0.2078, wR_2 = 0.3195
Largest diff. peak/hole / e Å^{-3}	0.56/-0.49

Crystal data and structure refinement for 3@DDQ.

Empirical formula	$C_{126}H_{124}Cl_8N_4O_{24}$
Formula weight	2361.88

Temperature/K	296.15
Crystal system	monoclinic
Space group	C2/c
a/Å	18.227(5)
b/Å	18.218(5)
c/Å	19.010(5)
α/°	90
β/°	111.022(4)
γ/°	90
Volume/Å^3	5892(3)
Z	2
ρ_{calc}g/cm^3	1.331
μ/mm^{-1}	0.265
F(000)	2472.0
Crystal size/mm^3	0.2 × 0.18 × 0.16
Radiation	MoKα (λ = 0.71073)
2Θ range for data collection/°	3.276 to 55.054
Index ranges	$-23 \le h \le 23$, $-23 \le k \le 23$, $-24 \le l \le 24$
Reflections collected	32617
Independent reflections	6693 [R_{int} = 0.0311, R_{sigma} = 0.0274]
Data/restraints/parameters	6693/85/434
Goodness-of-fit on F^2	1.041
Final R indexes [I>=2σ (I)]	R_1 = 0.0801, wR_2 = 0.2302
Final R indexes [all data]	R_1 = 0.1149, wR_2 = 0.2671
Largest diff. peak/hole / e Å^{-3}	0.85/-0.39
